# Kaempferol Attenuates Spaceflight‐Associated Knee Cartilage Degradation by Targeting NOX4‐Mediated Mitochondrial Dysfunction

**DOI:** 10.1002/advs.76477

**Published:** 2026-07-09

**Authors:** Yuesong Yin, Ruiling Xu, Meagan J. Makarczyk, Jia‐Jun Liu, Silvia Liu, Matthew Z. Shi, Roberta Di Carlo, Michael F. Almeida, Alex Garris, Sarah Day, Rebekah Sanchez‐Hodge, Jonathan C. Schisler, Aleeza H. Zilberman, Noah G. Allen, Angela J. Kubik, Elizabeth A. Blaber, Arnold Z. Olali, Wanqing Xie, Douglas C. Wallace, Christopher E. Mason, Peter G. Alexander, Giuseppe Intini, Afshin Beheshti, Hang Lin

**Affiliations:** ^1^ Department of Orthopaedic Surgery University of Pittsburgh School of Medicine Pittsburgh Pennsylvania USA; ^2^ Department of Bioengineering University of Pittsburgh Swanson School of Engineering Pittsburgh Pennsylvania USA; ^3^ Department of Pharmacology and Chemical Biology University of Pittsburgh School of Medicine Pittsburgh Pennsylvania USA; ^4^ Organ Pathobiology and Therapeutics Institute University of Pittsburgh School of Medicine Pittsburgh Pennsylvania USA; ^5^ Department of Periodontics and Preventive Dentistry University of Pittsburgh School of Dental Medicine Pittsburgh Pennsylvania USA; ^6^ Center For Craniofacial Regeneration University of Pittsburgh School of Dental Medicine Pittsburgh Pennsylvania USA; ^7^ McAllister Heart Institute and Department of Pharmacology The University of North Carolina At Chapel Hill Chapel Hill North Carolina USA; ^8^ Shirley Ann Jackson Ph.D. Center for Biotechnology and Interdisciplinary Studies and Department of Biomedical Engineering Rensselaer Polytechnic Institute Troy New York USA; ^9^ Center For Mitochondrial and Epigenomic Medicine The Children's Hospital of Philadelphia Philadelphia Pennsylvania USA; ^10^ The Division of Human Genetics The Department of Pediatrics The Perelman School of Medicine University of Pennsylvania Philadelphia Pennsylvania USA; ^11^ Department of Physiology and Biophysics Weill Cornell Medicine New York New York USA; ^12^ Orland Bethel Family Musculoskeletal Research Center University of Pittsburgh School of Medicine Pittsburgh Pennsylvania USA; ^13^ University of Pittsburgh UPMC Hillman Cancer Center Pittsburgh Pennsylvania USA; ^14^ Center For Space Biomedicine University of Pittsburgh School of Medicine Pittsburgh Pennsylvania USA; ^15^ McGowan Institute for Regenerative Medicine University of Pittsburgh School of Medicine Pittsburgh Pennsylvania USA; ^16^ Department of Surgery University of Pittsburgh School of Medicine Pittsburgh Pennsylvania USA; ^17^ Department of Computational and Systems Biology University of Pittsburgh School of Medicine Pittsburgh Pennsylvania USA; ^18^ Stanley Center for Psychiatric Research Broad Institute of MIT and Harvard Cambridge Massachusetts USA

**Keywords:** cartilage degradation, chondrocytes, kaempferol, microgravity, mitochondrial dysfunction, NOX4, osteoarthritis, senescence, spaceflight

## Abstract

Microgravity and space radiation experienced during spaceflight have adverse effects on musculoskeletal health, yet their impact on articular cartilage has not been fully understood. In this study, we demonstrated that simulated spaceflight on Earth leads to cartilage degradation in the knees of mice. Similar changes were also observed in mice exposed to actual spaceflight. To investigate mechanisms underlying spaceflight‐associated cartilage loss, human chondrocytes were encapsulated in a hydrogel scaffold and subjected to rotary culture to simulate microgravity‐induced alterations. Simulated microgravity increased the expression of biomarkers related to inflammation and cellular senescence. Additionally, rotary culture decreased mitochondrial respiration and increased reactive oxygen species production. Through RNA sequencing and bioenergetic profiling, we identified NADPH oxidase 4 (NOX4) as a crucial factor driving these changes. Moreover, kaempferol, a naturally occurring flavonoid that directly binds to NOX4, was found to partially reverse the harmful effects of microgravity on chondrocytes. Finally, systemic administration of kaempferol reduced cartilage degradation in mice subjected to simulated spaceflight on the ground. These findings establish that NOX4‐mediated mitochondrial dysfunction is a key mechanism underlying spaceflight‐induced cartilage degradation and highlight kaempferol as a potential protective measure for joint health in space.

## Graphical abstract

We have shown that both simulated and actual spaceflight contribute to cartilage degradation in the knees of mice. Using RNA sequencing and bioenergetic profiling, we identified NADPH oxidase 4 (NOX4) as a key factor driving these changes. Additionally, we found that kaempferol, a naturally occurring flavonoid that binds directly to NOX4, reduces cartilage degradation in mice exposed to simulated spaceflight.

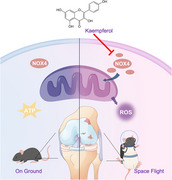

## 1 Introduction

Space travel plays a crucial role in advancing human exploration of the universe, particularly using crewed spacecraft for in‐depth exploration within the solar system. However, spaceflight also presents a unique environment characterized by microgravity, space radiation, isolation, and hostile/closed conditions, which significantly impact human physiology, including loss of bone density and muscle mass and increased inflammation [1, 2]. Systematic research on preventing and mitigating risks to human health is crucial for the sustainable advancement of future deep space exploration.

The knee joint is a crucial weight‐bearing joint in the human body, and changes in the mechanical properties of its articular surface are considered one of the key factors in the development of knee osteoarthritis (OA) [3]. OA is a chronic disease characterized by articular cartilage degradation, subchondral bone remodeling, synovitis, meniscal degeneration, and ligament changes [4]. At the cellular level, OA is characterized by hypertrophic chondrocyte buildup, extracellular matrix degradation and remodeling, increased inflammatory and oxidative stress signaling, expression of cellular senescence markers, as well as systemic mechanical instability and impaired tissue repair [4].

OA development and progression from the initial damage to pathological changes may take decades. Daily diurnal mechanical loading plays a crucial role in maintaining joint homeostasis. Studies have shown that integrated mechanical loading and dynamic fluid flow are essential for sustaining disc cell health and matrix homeostasis, which may similarly apply to articular cartilage under physiological conditions [5]. Mechanical loading also critically influences OA risk. For instance, low‐intensity, high‐frequency, and short‐duration mechanical loading promotes cartilage growth, while high‐intensity, low‐frequency, and long‐duration loading promotes cartilage loss [6]. Given the critical role of mechanics in knee joints, microgravity in space and the resultant loss of loading may increase the risk of cartilage degradation and the development of OA.

To date, research has not examined the association between prolonged exposure to space and OA pathogenesis in humans. However, increased circulating concentrations of urinary C‐terminal crosslinked telopeptide of type II collagen (CTX II), a biomarker of cartilage degradation [7, 8], were reported in astronauts post‐flight [9]. Animal studies on this topic are also limited, but maintaining mice in the International Space Station National Laboratory (ISS‐NL) for 35 days was sufficient to induce an arthritic‐like phenotype in knee articular cartilage [10]. Several on‐ground simulation experiments have specifically examined the impact of microgravity combined with radiation on knee joints. For example, Willey et al. investigated the effects of hindlimb unloading (HLU) and 1 Gy of total‐body x‐ray irradiation (IR) in rats. By day 13, rats exposed to both HLU and IR showed reduced glycosaminoglycan content and increased expression of matrix metalloproteinase‐13 (MMP‐13) relative to controls [11, 12]. Studies using in vitro culture systems have also reported the loss of cartilage matrix in microgravity [13]. A recent study further showed that microgravity induced metabolic shifts in cultured chondrocytes that were consistent with early OA metabolomic profiles in human synovial fluid [14]. These findings imply that long‐term spaceflight may increase the risk of developing OA [15, 16].

Currently, the mechanisms underlying knee joint degeneration in response to microgravity remain unclear. Consequently, there are no demonstrated methods to prevent or reverse spaceflight‐related cartilage loss. Research on astronaut health and model organisms has revealed multiple hallmarks of aging during spaceflight exposure, including DNA damage, mitochondrial dysregulation, and oxidative stress [1, 17, 18]. Specifically, we conducted a comprehensive multi‐omics analysis and identified mitochondrial dysfunction as a unifying driver of the physiological effects of spaceflight [19].

In this study, we hypothesized that exposure to spaceflight conditions induces mitochondrial dysfunction and cartilage degradation, and that preserving mitochondrial function protects against cartilage loss. To test the hypothesis, we investigated the impact of space travel on the knee joint in mice and in primary human chondrocytes cultured in a rotary cell culture system (RCCS) to simulate microgravity in vitro. Particularly, we performed comprehensive transcriptomic profiling, histological staining, and biomechanical evaluation to elucidate the molecular and biomechanical mechanisms underlying microgravity‐induced cartilage degradation. To explore potential countermeasures, we evaluated the efficacy of kaempferol (KMP), a natural flavonoid with well‐documented mitochondrial protective properties, including reducing mitochondrial reactive oxygen species (ROS) production, preserving mitochondrial membrane potential, and supporting cellular energy metabolism under stress conditions [20, 21, 22]. More importantly, KMP has also demonstrated efficacy in reducing OA progression in rat models [23]. Given that mitochondrial dysfunction is a key contributor to cartilage degradation [24] and underlies many other spaceflight‐related health risks [25], kaempferol was expected to mitigate the detrimental impact of microgravity on cartilage.

## 2 Results

### 2.1 Both Ground‐Based Microgravity Simulations and Actual Spaceflight Induce Cartilage Degradation in Mice

**FIGURE 1:**
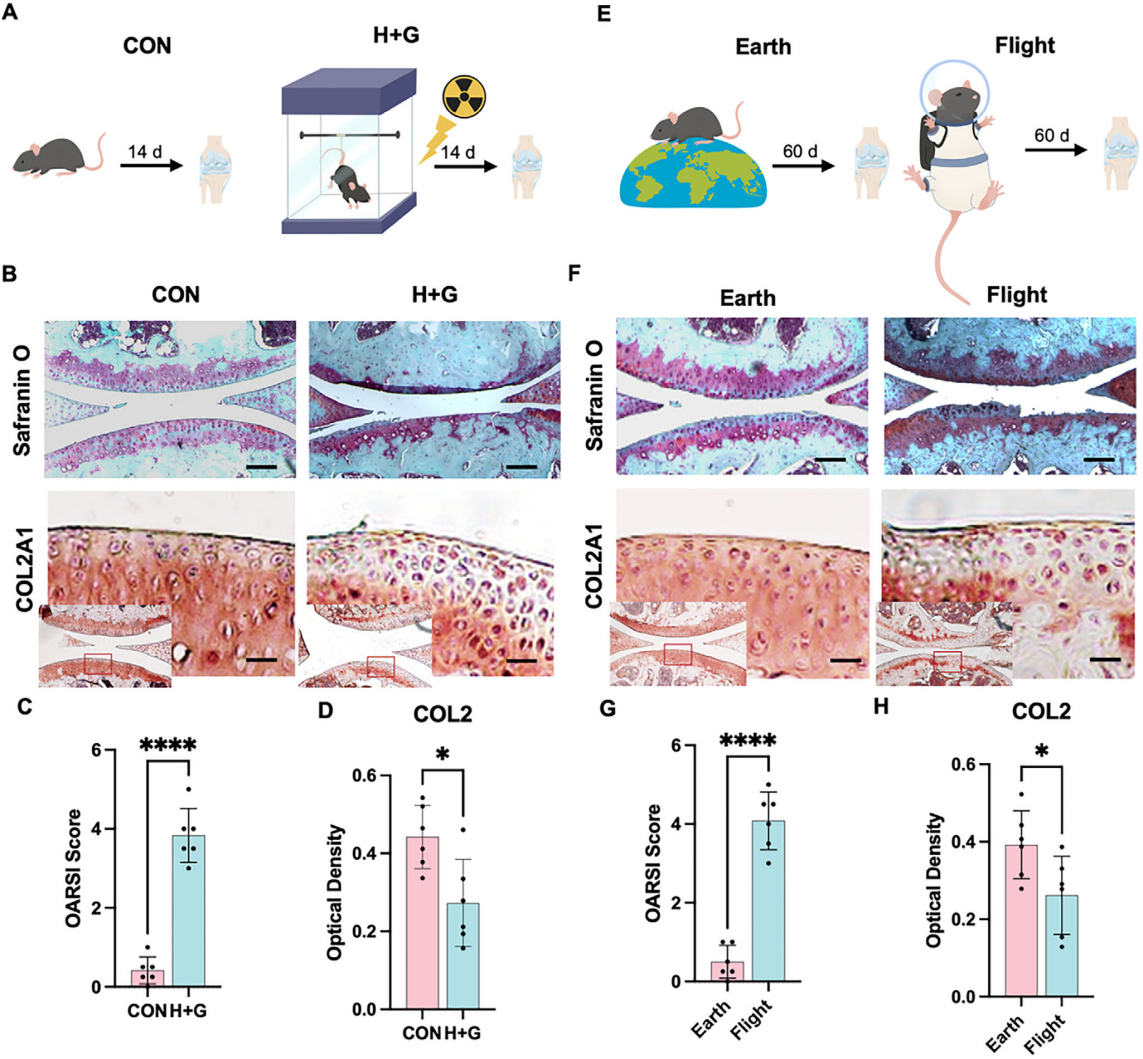
Simulated microgravity and spaceflight induce cartilage loss in the murine knee joint. (A, E) Schematic illustration of the experimental design for (A) simulated microgravity (hindlimb unloading combined with galactic cosmic ray (H+G) and (E) spaceflight. (B, F) Representative Safranin O/Fast Green and collagen type II‐A1 (COL2A1) immunohistochemical staining (IHC) of knee joint sections from (B) control (CON) and H+G mice, or (F) mice subjected to spaceflight (Flight) and their on‐Earth control (Earth). Scale bar: 100 µm in Safranin O staining and 25 µm in COL2A1 IHC. Red boxes in lower‐magnification images indicate the locations of higher‐magnification images. (C, G) Quantification of cartilage degradation using the OARSI histological score. (D, H) Semi‐quantitation of COL2A1 staining. *n* = 6 mice per group. Data are presented as mean ± SD. ^***^ *p* < 0.001; ^****^ *p* < 0.0001. Statistics were conducted with an unpaired two‐tailed Student's t‐test.

As illustrated in Figure 1A,E, mice subjected to simulated microgravity and actual spaceflight were included in this study. The sagittal section was used, and data from the medial compartment were shown throughout the study. Safranin O staining demonstrated a marked reduction in glycosaminoglycan (GAG) content in both the simulated and actual spaceflight groups compared with their respective controls (Figure 1B,F). In addition, evident structural damage of the articular cartilage surface was observed, including surface fibrillation and focal cartilage defects extending toward the subchondral bone. Histological evaluation using the OARSI scoring system confirmed significant cartilage degradation in mice undergoing either simulated or actual spaceflight (Figure 1C,G). Immunohistochemical (IHC) staining for type II collagen‐α1 (COL2A1) showed a substantial decrease in COL2A1 levels in both experimental groups compared with their respective controls (Figure 1B,D,F,H). We also examined other proteins related to cartilage quality. Specifically, COL1A1 and MMP‐13 levels were markedly increased in both the simulated microgravity (H+G) and spaceflight (Flight) groups (Figure S1). In contrast, COL10A1 levels were low in all groups. Collectively, these findings suggest that the spaceflight environment, whether simulated or actual flight, significantly promotes cartilage degradation in the mouse knee joint.

### 2.2 Rotary Culture‐Simulated Microgravity Induces Degradation of Engineered Cartilage

**FIGURE 2:**
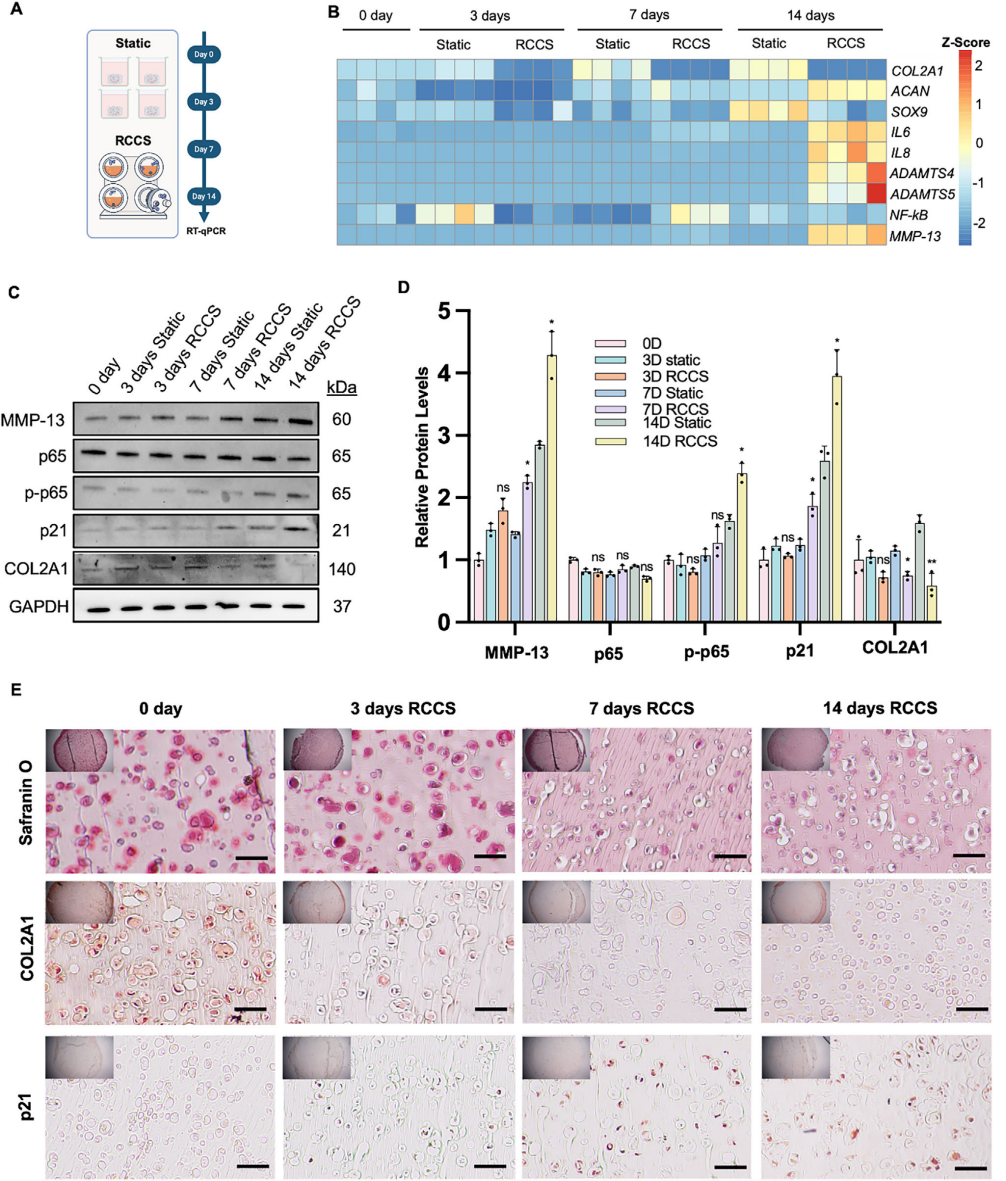
Simulated microgravity impairs chondrocyte phenotype and promotes inflammation and matrix degradation. (A) Schematic illustration of the study. RCCS=rotary cell culture system. (B) Heatmap of gene expression (Z‐score normalized) from qRT‐PCR analysis of chondrocytes cultured under static or RCCS conditions for 3, 7, and 14 days. *n* = 4 constructs per group. (C) Western blot to examine the levels of selected proteins in chondrocytes subjected to static or RCCS culture. GAPDH was used as the loading control. (D) Semi‐quantification of western blot band intensities normalized to GAPDH and expressed relative to the day 0 group. *n* = 3 constructs per group. Data are shown as mean ± SD. One‐way ANOVA followed by Holm–Šídák's multiple comparisons test was performed. *, statistically different between static and RCCS group; ns, no significant difference. (E) Representative imaging from Safranin O/Fast Green staining, and COL2A1 and p21 IHC. Scale bars: 10 µm. Abbreviations: SOX9, SRY‐box transcription factor 9; NF‐kB, nuclear factor kappa B.

We maintained human mesenchymal stromal cell (hMSC)‐derived cartilage tissue in a rotary cell culture system (RCCS) to simulate microgravity in vitro (Figures 2A and S2). After 14 days of rotary culture, chondrocytes exhibited a pronounced inflammatory phenotype, characterized by significant upregulation of interleukin‐6 (IL6) and interleukin‐8 (IL8) (Figures 2B and S3). In addition, expression levels of matrix metallopeptidase 13 (*MMP‐13*), ADAM metallopeptidase with thrombospondin type 1 motif (ADAMT)‐4 and 5, were markedly increased in the microgravity group compared to the control group, suggesting elevated catabolic activities. Interestingly, while *COL2A1* expression was downregulated, aggrecan (*ACAN*) expression was upregulated. Western blot analysis further confirmed the findings from qRT‐PCR. Specifically, after 14 days of simulated microgravity, chondrocytes showed increased MMP13 and phosphorylated p65 protein levels, along with decreased COL2A1 levels (Figure 2C,D). Interestingly, higher p21 levels were also observed in cartilage under microgravity. Consistent with these molecular changes, histological analyses revealed that chondrocytes cultured under simulated microgravity for 14 days showed a marked reduction in GAG content, decreased COL2A1 staining, and increased p21 expression compared with the static control groups (Figure 2E and Figure S4). Taken together, simulated microgravity via RCCS induces inflammation in chondrocytes and impaired matrix integrity.

### 2.3 Microgravity Induces Mitochondrial Dysfunction

**FIGURE 3:**
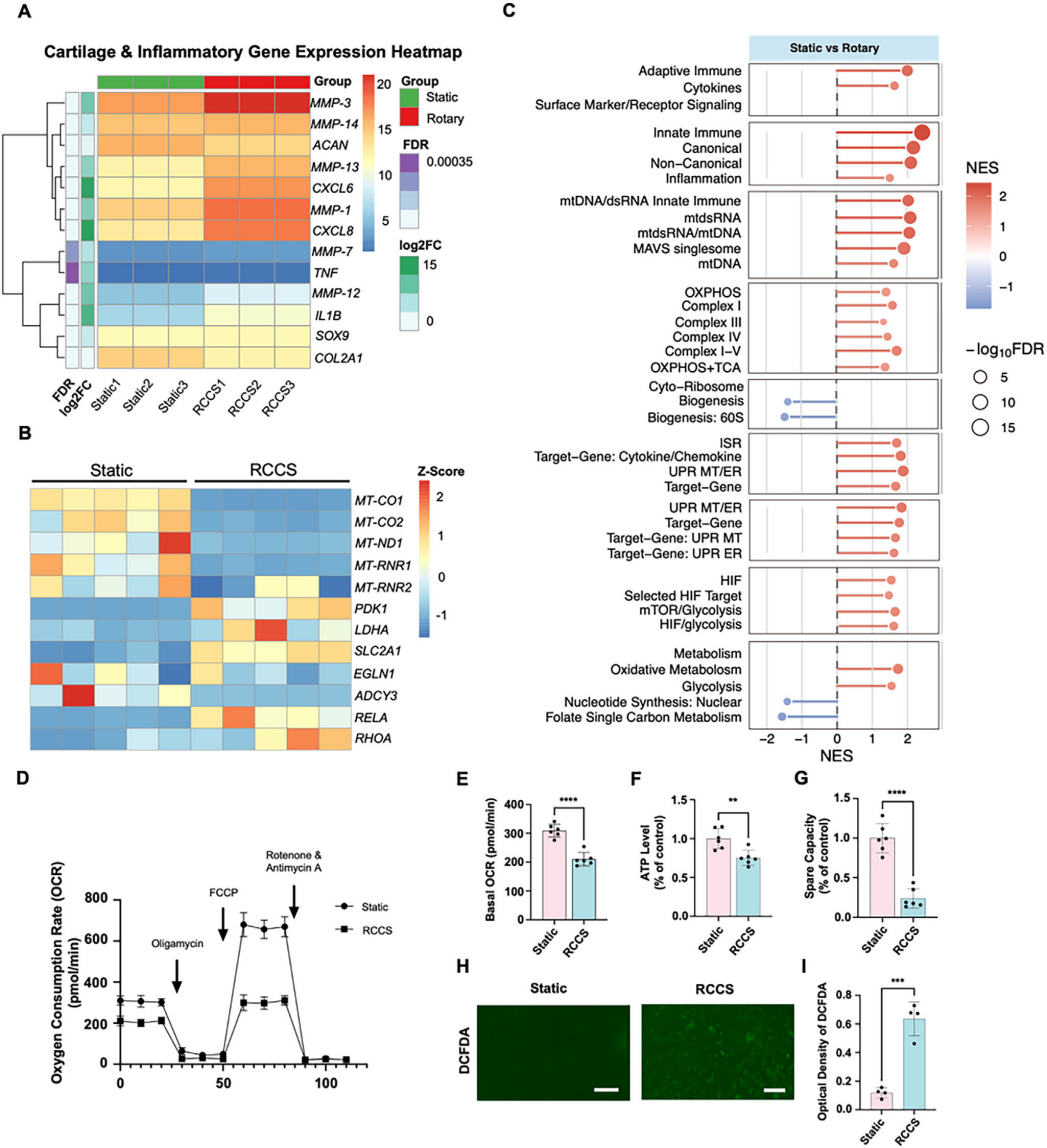
Simulated microgravity induces mitochondrial dysfunction in cultured chondrocytes. (A) Heatmap of expression levels of representative chondrogenic and inflammation‐related genes from RNA‐seq data. (B) Heatmap of mitochondria‐related gene expression across two culture conditions. Data were from qRT‐PCR analysis. (C) Bubble plot showing significantly enriched pathways in static versus rotary conditions. Circle size represents −log10 adjusted *p*‐value, and color indicates normalized enrichment score (red = enriched in rotary, blue = enriched in static). (D) Mitochondrial stress test using Seahorse XF analysis. (E–G) Quantification of basal Oxygen Consumption Rate (OCR) (E), intracellular ATP levels (F), and spare respiratory capacity (G) (*n* = 3 per group). (H, I) DCFDA staining and quantification. *n* = 5 per group. Scale bars: 20 µm in H. Data are presented as mean ± SD. ^**^ *p* < 0.01; ^***^p < 0.001; ^****^ *p* < 0.0001. Abbreviations: MT‐CO1, mitochondrially encoded cytochrome c oxidase I; MT‐CO2, mitochondrially encoded cytochrome c oxidase II; MT‐ND1, mitochondrially encoded NADH dehydrogenase 1; MT‐RNR1, mitochondrially encoded 12S RNA; MT‐RNR2, 16S rRNA, mitochondrial; PDK1, pyruvate dehydrogenase kinase 1; LDHA, lactate dehydrogenase A; SLC2A1, solute carrier family 2 member 1; EGLN1, egl‐9 family hypoxia inducible factor 1; ADCY3, adenylate cyclase 3; RELA, RELA proto‐oncogene, NF‐kB subunit; RHOA, ras homolog family member A.

To further delineate the impact of simulated microgravity on chondrocytes, we performed RNA‑sequencing (RNA‐seq) on chondrocytes cultured for 14 days in RCCS. Cells in static culture were used as the control. The transcriptomic data demonstrated a significant difference between the RCCS and Static groups (Figure S5A). Particularly, marked upregulation of inflammatory and senescence signatures was observed, accompanied by downregulation of chondrogenic markers in the RCCS group, findings that corroborate our previous observations (Figure 3A). Interestingly, gene‑set enrichment analysis of the RNA‑seq results showed that differentially expressed genes were predominantly clustered in mitochondrial‑related biological processes (Figure S5B). KEGG pathway enrichment further revealed multiple signaling cascades relevant to chondrocyte metabolism, with prominent representation of the Hypoxia‐inducible factor 1 (HIF‑1), apoptosis, and PI3K–Akt pathways, all closely linked to mitochondrial function (Figure S5B).

Transcriptomic analysis suggested that impaired mitochondrial metabolism may underlie microgravity‐induced cartilage damage (Figure S5B). Therefore, we performed qRT‐PCR to assess the expression levels of key genes regulating mitochondrial function. Under RCCS culture, chondrocytes exhibited significant up‑regulation of *PDK1, LDHA, SLC2A1, RELA*, and *RHOA*, whereas genes essential for oxidative phosphorylation, ATP synthesis, and mitochondrial translation were down‑regulated (Figure 3B and Figure S5C–N). Notably, the RNA‑seq data corroborated the results from qRT‐PCR (Figure S6A). Pathway enrichment analysis (Figure 3C and Figure S6B–F) revealed that multiple mitochondrial processes, including oxidative phosphorylation and oxidative metabolism, were significantly suppressed under RCCS conditions, further highlighting mitochondrial dysfunction as a central mechanism driving microgravity‐induced cartilage loss.

Seahorse assay further revealed that 14 days of RCCS exposure led to pronounced decreases in basal oxygen consumption rate, ATP‑linked respiration, and spare respiratory capacity, indicating suppressed mitochondrial function (Figure 3D–G). Consistently, DCFDA staining demonstrated a significant increase in mitochondrial reactive oxygen species (ROS) generation in RCCS cells compared with static controls (Figure 3H,I). In summary, these results indicate that microgravity induces mitochondrial dysfunction in chondrocytes.

### 2.4 Kaempferol Plays Protective Roles in Chondrocytes Subjected to Microgravity

Identifying pharmacological agents that counteract microgravity‑induced chondrocyte injury is essential for protecting the knee joint during spaceflight. KMP has been previously reported to ameliorate mitochondrial damage [20] and to exert chondroprotective effects [26]. We, therefore, evaluated the potential of KMP to mitigate microgravity‑induced cartilage degradation. Specifically, IL‑1β‐stimulated chondrocytes were treated with a concentration gradient of KMP (2.5, 5, 10,‐20, and 40 µM). CCK‑8 assays indicated that KMP exhibited no cytotoxicity across all tested doses (Figure S7A). qRT‐PCR analysis revealed concentration‑dependent improvements in chondrogenic markers and reductions in the expression of proinflammatory genes (Figure S7B–J). Based on the KMP‑induced elevation of *COL2A1* expression, the half‑maximal effective concentration (EC_50_) was calculated to be 20 µM (Figure S7K), which was selected as the working concentration for subsequent in vitro experiments.

**FIGURE 4:**
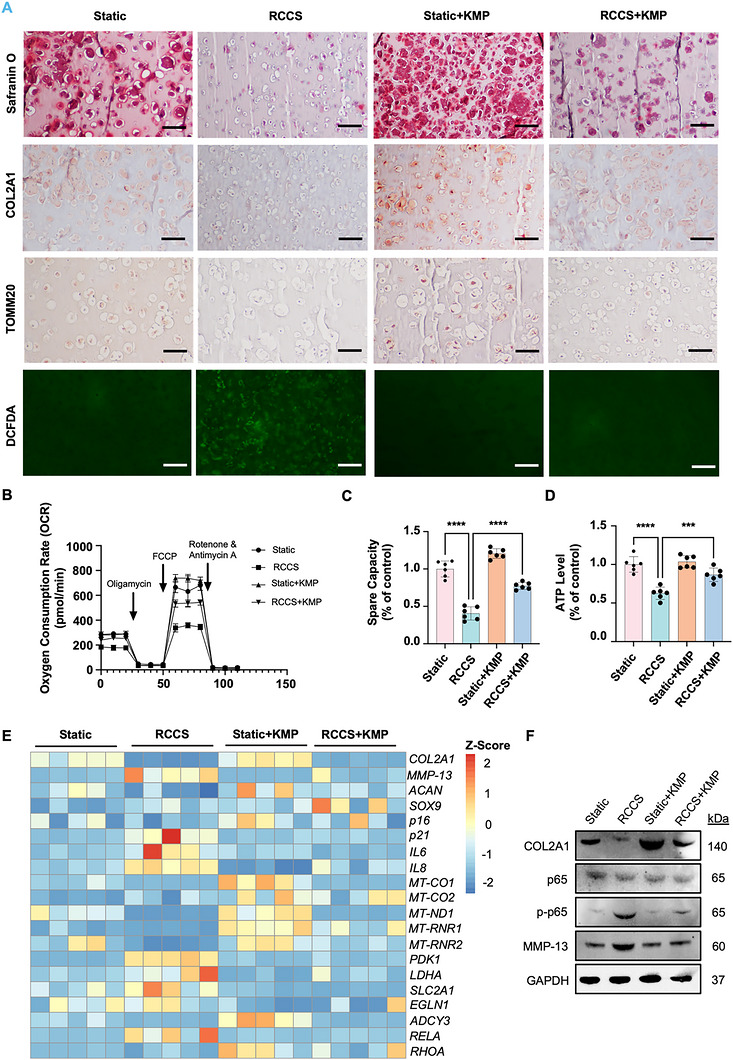
Kaempferol mitigates microgravity‐induced mitochondrial dysfunction and cartilage degradation. (A) Representative images from Safranin O/Fast Green staining, COL2A1 and TOMM20 IHC, and DCFDA fluorescence in chondrocyte‐laden hydrogels cultured under static or rotary (RCCS) conditions, with or without kaempferol (KMP, 20 µM) treatment. Scale bars: 10 µm in Safranin O staining and IHC, 20 µm in DCFDA staining. (B) Mitochondrial stress test showing oxygen consumption rate (OCR) traces from Seahorse XF analysis under different culture conditions. (C, D) Quantification of spare respiratory capacity (C) and intracellular ATP levels (D); *n* = 6 constructs per group. One‐way ANOVA followed by Holm‐Šídák's multiple comparisons test was performed. Data are presented as mean ± SD. ^***^ *p* < 0.001; ^****^ *p* < 0.0001. (E) Heatmap of expression levels of selected genes related to chondrogenesis, inflammation, and mitochondrial function; *n* = 5 constructs per group. (F) Western blot to examine protein levels in different groups. GAPDH served as the loading control.

After 14 days of RCCS culture, safranin O staining showed that KMP partially reversed RCCS‐induced GAG loss. Immunohistochemistry (IHC) further revealed that KMP mitigated the losses of COL2A1 and mitochondrial mass (TOMM20) (Figure 4A and Figure S8A,B). Consistent with this, DCFDA staining demonstrated a significant decrease in mitochondrial ROS following KMP treatment under RCCS conditions (Figure 4A and Figure S8C). Bioenergetic profiling indicated that RCCS decreased basal oxygen consumption rate, spare respiratory capacity, and ATP production, effects partially rescued by KMP treatment (Figures 4B–D and Figure S8D). qRT‐PCR and Western blot analyses confirmed that KMP treatment counteracted the RCCS‑induced down‐regulation of chondrogenic markers and the up‐regulation of inflammatory and senescence‑associated genes (Figure 4E,F, and Figures S8E, and S9A–T).

We also evaluated the effects of simulated microgravity and KMP treatment using OA‐like cells, which were generated from senescent hMSCs as we reported before [27]. In general, microgravity also causes detrimental effects on OA chondrocytes, while KMP partially reversed the changes (Figure S10). Collectively, these data suggest that KMP is a robust agent for attenuating microgravity‑induced mitochondrial dysfunction and cartilage degradation.

### 2.5 Kaempferol Functions Partially Through Targeting NOX4

**FIGURE 5:**
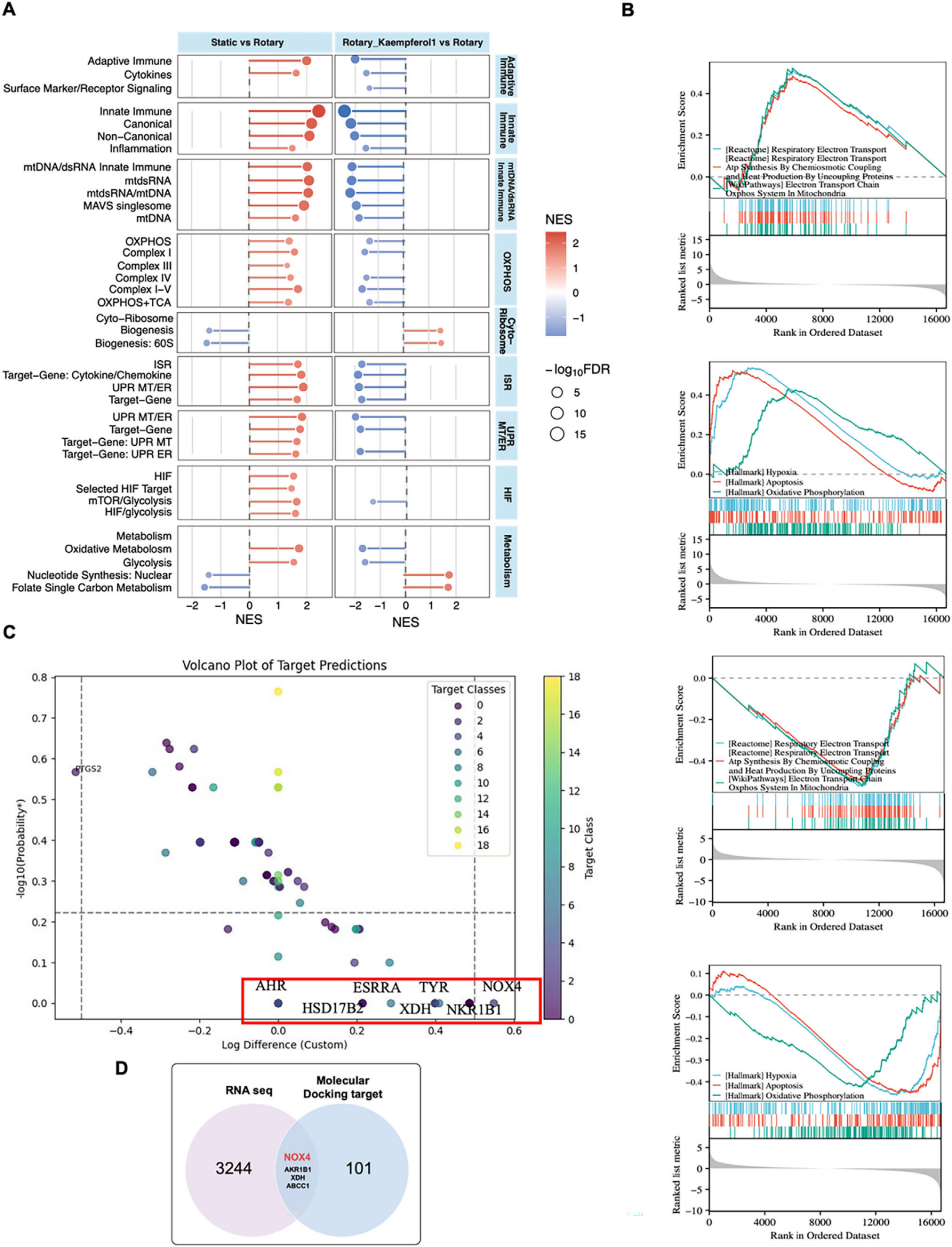
Transcriptomic and computational analyses identify NOX4 as a key target of kaempferol. (A) Bubble plots of pathway enrichment analysis comparing static versus rotary culture (left) and rotary + kaempferol versus rotary (right). Circle size indicates −log10 adjusted *p*‐value, and color represents normalized enrichment score (NES). Rotary culture suppressed oxidative phosphorylation and metabolism while activating immune/inflammatory pathways; kaempferol treatment reversed these changes. (B) Gene set enrichment analysis (GSEA) plots reveal that kaempferol reverses rotary culture‐induced activation of oxidative phosphorylation, apoptosis, and hypoxia pathways. (C) Volcano plot of predicted kaempferol‐binding targets (based on molecular docking). Highlighted candidates include NOX4, XDH, AKR1B1, and ABCC1, all of which show significant predicted binding affinity. (D) Overlap between predicted docking targets (*n* = 101) and RNA‐seq DEGs (*n* = 3244) identifies NOX4 as the most promising shared candidate mediating kaempferol's protective effects.

To elucidate the molecular target(s) of kaempferol action, we performed RNA‐seq on chondrocytes cultured in the RCCS system with or without KMP treatment. Principal‐component analysis confirmed that KMP shifted the microgravity transcriptome toward the static control profile (Figure S11A). A pairwise comparison of DEGs between RCCS versus Static and KMP + Rotary versus Rotary identified 3244 DEGs (Figure S11B). Consistently, pathway enrichment analysis using bubble plots revealed that rotary culture activated immune and inflammatory signaling while suppressing oxidative phosphorylation and metabolic pathways. In contrast, KMP treatment largely reversed these changes, restoring mitochondrial function and dampening aberrant immune responses (Figure 5A and Figure S11C–K). In line with this, gene‐set enrichment analysis further showed that KMP counteracted the rotary culture‐induced activation of oxidative phosphorylation, hypoxia, and apoptosis pathways, reinstating a homeostatic expression pattern (Figure 5B).

Reverse‐docking against the Protein Data Bank (PDB) yielded 101 putative KMP‐binding proteins, seven of which displayed high predicted affinity (Figure 5C). Cross‐referencing these 101 proteins with the intersecting DEGs highlighted four genes, including NADPH oxidase 4 (*NOX4*), aldo‐keto reductase family 1 member B (*AKR1B1*), xanthine dehydrogenase (*XDH*), and ATP‐binding cassette subfamily C member 1 (*ABCC1*), that were both differentially expressed and computationally predicted KMP targets. Among them, NOX4, a ROS‐generating enzyme, emerged as the most compelling candidate owing to its sub‐mitochondrial localization and strong predicted KMP affinity (Figure 5D), and was thus selected for further examination.

**FIGURE 6:**
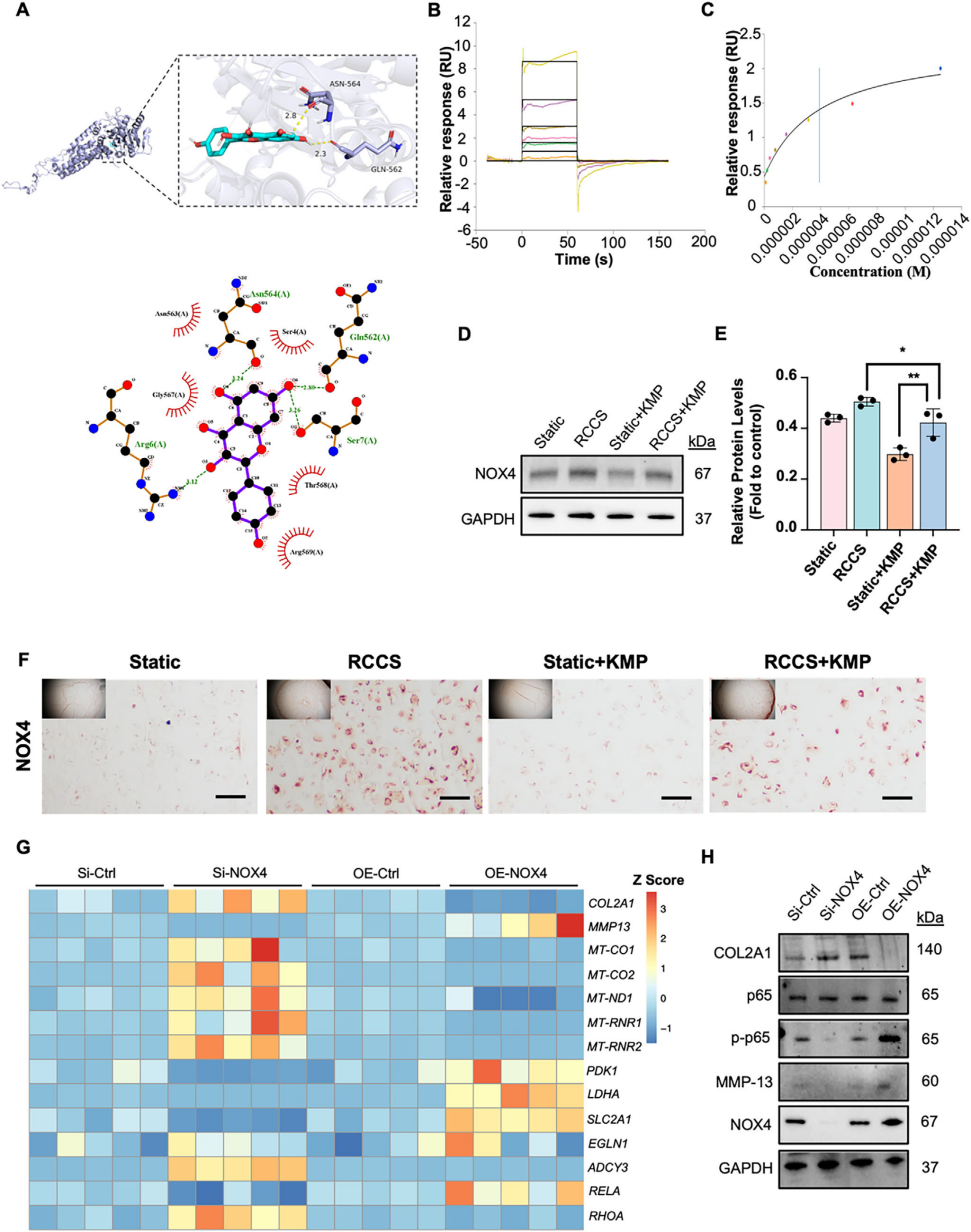
KMP directly binds and inhibits NOX4 to preserve mitochondrial and cartilage homeostasis. (A) Molecular docking model showing KMP binding within the catalytic pocket of NOX4 with favorable hydrogen bonding and hydrophobic interactions. (B) Surface plasmon resonance (SPR) sensorgram depicting concentration‐dependent binding of KMP to immobilized NOX4 protein. (C) The binding curve from the SPR analysis shows a dissociation constant of ∼3.9 µM, indicating moderate‐to‐strong affinity. (D, E) Western blot and quantification (*n* = 3 per group). Data are presented as mean ± SD. ^*^ *p* < 0.05; ^**^ *p* < 0.01. GAPDH was used as the loading control. (F) NOX4 IHC. Scale bars: 10 µm. (G) Heatmap of expression levels of selected genes in chondrocytes treated with NOX4 siRNA (si‐NOX4) or lentiviral vectors carrying the NOX4 gene (OE‐NOX4). Scrambled siRNA (si‐Ctrl) and lentiviral vectors carrying a control gene (OE‐Ctrl) were used as the control, respectively. (H) Western blot to examine protein levels. GAPDH was used as the loading control.

To confirm a direct interaction between KMP and NOX4, we first performed molecular docking with AutoDock Vina. KMP fitted tightly into the NOX4 catalytic pocket with a binding energy of −7.4 kcal mol^−^^1^, indicative of spontaneous binding under physiological conditions (Figure 6A and Figure S12A–D). NOX4 immobilized on a CM5 chip can bind KMP with an affinity constant of 3.92e‐06 M as determined in an SPR assay (Figure 6B,C). Results from western blot and qRT‐PCR demonstrated that RCCS upregulated NOX4 expression, whereas KMP treatment significantly blunted this induction (Figure 6D,E). IHC for NOX4 demonstrated a similar finding (Figure 6F and Figure S13). Functional assays further substantiated NOX4 as the critical KMP target. Specifically, silencing NOX4 (si‑NOX4) reduced inflammatory gene expression and enhanced chondrogenic markers, while exogenous recombinant NOX4 negated the protective effects of KMP, exacerbating inflammation and impairing chondrogenesis (Figure 6G and Figure S14A–N). Western blot analysis confirmed the findings from qRT‐PCR (Figure 6H and Figure S14O). Collectively, these data indicate that KMP protects mitochondrial function and cartilage integrity by directly binding to and suppressing NOX4, thereby limiting mitochondrial ROS accumulation.

### 2.6 Kaempferol Protects Cartilage in Mice Subjected to Simulated Spaceflight

**FIGURE 7:**
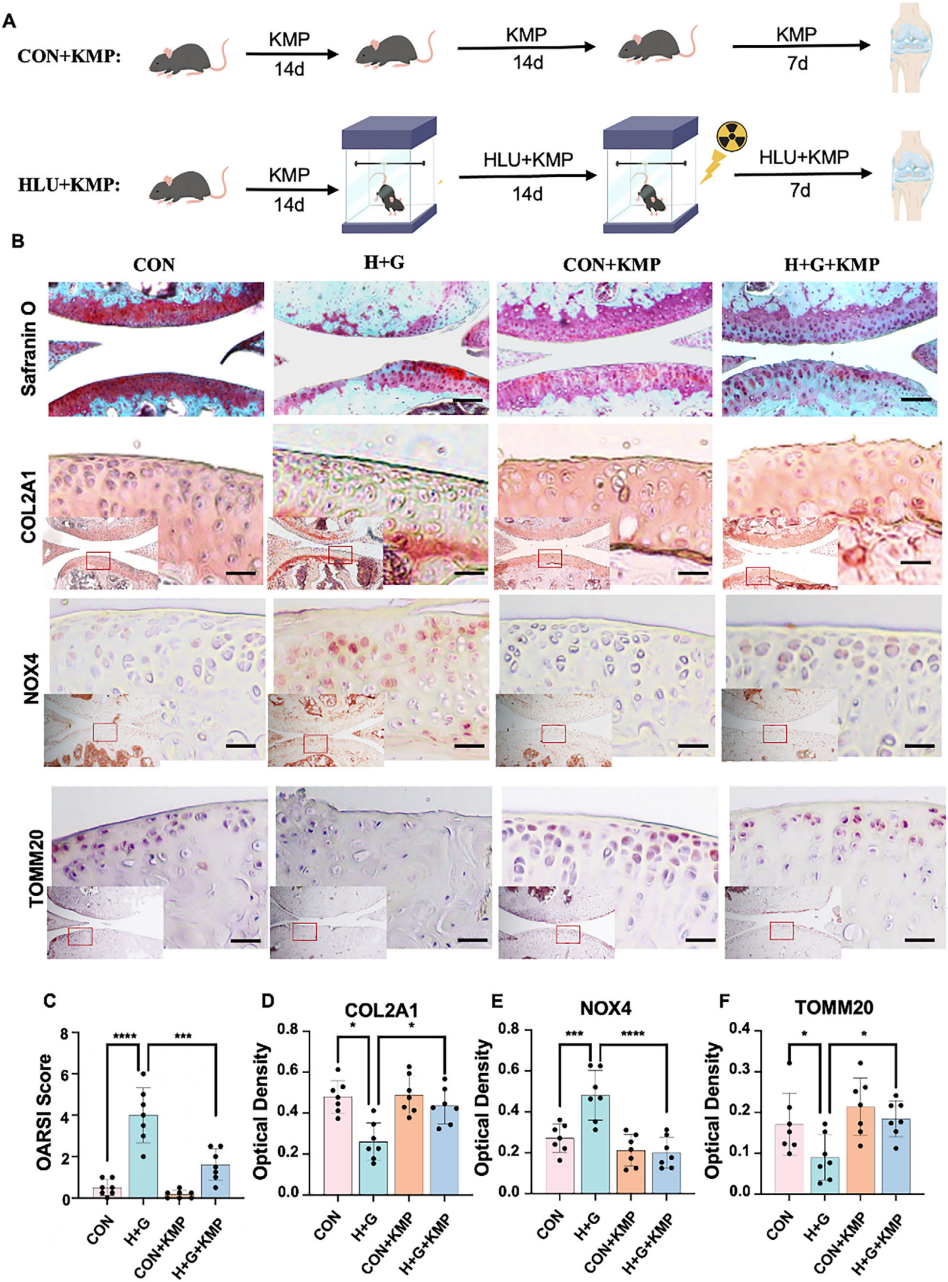
Kaempferol protects spaceflight‐induced cartilage degradation in mice. (A) A schematic illustration of the experimental design for KMP treatment with either control (CON) or simulated microgravity (hindlimb unloading (HLU) combined with galactic cosmic ray, (H+G)). (B) Representative imaging of knee joint sections from CON, H+G, kaempferol‐treated control (CON+KMP), and kaempferol‐treated spaceflight simulation (H+G+KMP) groups. Sections were stained with Safranin O/Fast Green for cartilage matrix, and IHC was performed for COL2A1, NOX4, and TOMM20. Red boxes in lower‐magnification images indicate the locations of higher‐magnification images. (C) OARSI scores. Scale Bar: 100 µm in Safranin O staining and 25 µm in IHC. (D–F) Semi‐quantification of (D) COL2A1, (E) NOX4, and (F) TOMM20 levels based on the IHC imaging (*n* = 6–8 per group). One‐way ANOVA followed by Holm–Šídák's multiple comparisons test was performed. Data are shown as mean ± SD. ^***^ *p* < 0.001; ^****^ *p* < 0.0001.

Finally, we evaluated the potential of KMP in protecting cartilage from spaceflight‐associated damage by orally administering the compound to mice subjected to on‐earth simulated spaceflight (Figure 7A). Safranin O staining and COL2A1 IHC showed that KMP markedly attenuated the microgravity‑induced loss of GAGs and COL2A1 (Figure 7B–D). Moreover, KMP treatment reduced NOX4 accumulation and restored mitochondrial abundance in articular cartilage, as evidenced by NOX4 and TOMM20 immunostaining (Figure 7B,E,F). Interestingly, KMP treatment also effectively suppressed COL1A and MMP‐13 expression in cartilage (Figure S15A–C). These results support that microgravity triggers cartilage degradation via mitochondrial dysfunction in chondrocytes. In contrast, KMP counteracts this process by binding to and inhibiting NOX4, thereby limiting ROS accumulation and preserving cartilage integrity.

## 3 Discussion

Consistent with previous spaceflight‐related studies [11, 12, 15], we observed that both mice subjected to 60 days of spaceflight aboard the International Space Station and those in ground‐based simulation models (radiation exposure combined with hindlimb unloading) displayed reduced GAG and COL2A1 content in the knee joint cartilage, accompanied by varying degrees of knee joint surface irregularity. These findings indicate that microgravity and radiation collectively disrupt cartilage homeostasis and impair matrix integrity.

Previous studies have demonstrated that the microgravity and radiation associated with space travel can adversely affect the musculoskeletal system, leading to muscle atrophy and decreased bone mineral density [28]. In hyaline cartilage, physiological mechanical stimulation, which is diminished during space flight, is required to maintain chondrocyte phenotype and function, and to secrete extracellular matrix components such as GAGs and COL2A1, thereby maintaining matrix stability and preserving knee joint structure [29]. For instance, joint immobilization in rats resulted in a significant decrease in cartilage thickness and cell density [30]. Kwok et al. showed that spaceflight and hind limb unloading induced an arthritic phenotype in rodent knee articular cartilage and menisci, supporting the idea that unloading directly impairs synovial joint homeostasis [10]. Meanwhile, another study showed that disuse atrophy of the articular cartilage and subchondral bone in the rat tibia induced by hindlimb suspension was partially reversed by physiological reloading [31]. These findings suggest that the absence of mechanical stimulation may play a central role in cartilage degradation due to spaceflight. In addition to COL2A and GAG content, the effects of microgravity on cartilage fibrosis‐ and hypertrophy‐related markers, to the best of our knowledge, have only been reported in in vitro models. For instance, short‐term simulated microgravity has been reported to reduce COL1 and COL10 expression in human chondrocytes, but other models, particularly stem cell or meniscus‐based systems, suggest that unloading may still disturb chondrogenic stability and promote hypertrophic remodeling in a context‐dependent manner [32, 33, 34]. In the current study, we observed the upregulation of COL1, not COL10, in native cartilage from rats subjected to microgravity. This inconsistency likely reflects the differences between in vitro models and animals. In future studies, the potential of spaceflight in promoting fibrous and hypertrophic conversions of chondrocytes requires further careful examination through other analytical methods, such as western blot or advanced magnetic resonance imaging.

The mechanism underlying the association between spaceflight and cartilage degradation remains poorly understood. An in vitro platform that simulates spaceflight is required for mechanistic studies and screening countermeasures. The RCCS is widely regarded as an effective in vitro model for simulating microgravity by maintaining cell aggregates in a continuous state of free fall through horizontal rotation [35]. A recent study showed that RCCS caused metabolic shifts in chondrocytes resembling the early OA‐associated metabolomic profiles observed in human synovial fluid [36] and induced the loss of cartilage matrix in human mesenchymal stem cells [13]. In chondrocytes, microgravity leads to OA‐like alterations characterized by metabolic reprogramming, particularly a reduction in anabolic pathways, pointing to a reduction in ECM synthesis [14]. In engineered cartilage, transcriptomic and proteomic profiling reveal changes resembling osteoarthritic degradation [37]. Therefore, we also employed RCCS to simulate microgravity in vitro, without radiation due to its DNA‐damaging effects [38], a change observed in aged and OA chondrocytes [39].

We observed a progressive decline in the chondrocyte phenotype with increasing rotation duration, and by day 14, a marked reduction in extracellular matrix components (GAGs and COL2A1) together with increased senescence and inflammatory markers (P21, IL‐6, IL‐8). These findings align with prior RCCS simulated microgravity studies in cartilage that reported OA‐like metabolic reprogramming in chondrocytes and matrix loss in MSC‐derived cartilage, along with the common pattern of COL2A1 downregulation and MMP‐13 upregulation [33]. MMP‐13 is a key mediator of cartilage catabolism because it preferentially degrades type II collagen; therefore, the increase in MMP‐13 observed under unloading‐related conditions may indicate activation of a bona fide matrix‐destructive program, which is in line with rodent unloading and spaceflight studies reporting elevated MMP‐13 alongside loss of glycosaminoglycans and other arthritic changes in synovial joint tissues [40]. Notably, while those cartilage studies seldom profiled canonical inflammatory cytokines, RCCS‐induced inflammatory activation has been independently demonstrated in other cell types. In endothelial cells, RCCS/SMG induced deleterious actin organization and increased expression of apoptotic proteins; osteoblasts exhibited increased IL‐6 under microgravity/radiation, and monocytes displayed impaired ability to resolve inflammation under RCCS, which aligns with our observed increase in the expression of IL‐6/IL‐8 [41, 42, 43]. Human spaceflight datasets have consistently reported heightened circulating proinflammatory cytokines, including IL‐6 and TNF‐α, during or after missions, reinforcing the translational relevance of an inflammatory component in microgravity‐associated cartilage degradation [44, 45]. Our RCCS model captured both matrix attrition (GAG/COL2A1 loss with literature‐concordant MMP‐13 remodeling) and inflammation/senescence axes, thereby offering an integrated platform to mechanistically interrogate microgravity‐driven cartilage aging and to benchmark countermeasures.

Previous studies have shown that microgravity can induce mitochondrial oxidative stress and dysfunction [46], ER stress [47], and DNA damage [48]. Da Silveira et al. conducted the first comprehensive multi‐omics analysis of astronauts and rodent models, revealing that mitochondrial stress, including altered oxidative phosphorylation and upregulated stress‐response pathways, is a central biological hub across tissues during space missions [25]. In our study, pathway enrichment analysis from RCCS‐exposed chondrocytes similarly emphasized mitochondria‐associated processes, including PI3K‐AKT, HIF‐1, and apoptosis, supporting the external validity of our model. Moreover, while features such as decreased membrane potential, lowered ATP production, and elevated ROS are well documented in OA chondrocytes [49], the alignment with spaceflight‐associated mitochondrial pathology strengthens the biological plausibility of our findings. Notably, RCCS simulates microgravity in a cartilage‐focused context and does not incorporate factors like radiation or systemic stress. Therefore, while our model captures a key mechanistic axis relevant to cartilage degradation, it remains a simplified representation of the complex physiological challenges experienced in true spaceflight.

Given the critical role of mitochondria in microgravity‐induced chondrocyte dysfunction, identifying effective strategies to prevent or mitigate cartilage damage has become a key focus of our research. Kaempferol, a naturally occurring flavonoid widely found in tea and various plants [50], has been reported to exert protective effects on mitochondria, including the attenuation of oxidative stress and reduction of intracellular ROS accumulation [51]. Consistent with this, KMP has demonstrated chondroprotective effects in osteoarthritis models, where it suppressed IL‐1β‐induced inflammatory mediators in rat chondrocytes by inhibiting NF‐κB signaling [52], and it has also been shown to delay aging‐related phenotypes in Drosophila by alleviating ER stress and modulating insulin signaling [53]. These terrestrial findings are further supported by spaceflight research showing that mitochondrial stress is a unifying driver of the biological consequences of microgravity and other space hazards [54]. In our study, KMP treatment on chondrocytes under RCCS effectively reduced mitochondrial ROS production, improved respiratory function, and reversed extracellular matrix degradation, inflammation, and senescence in chondrocytes. In vivo KMP administration partially restored GAG and COL2A1 content in the knee joint. Taken together, these results suggest that KMP, building on its established roles in OA and aging, holds promise as a mitochondria‐targeted countermeasure for spaceflight‐associated cartilage degradation.

We performed RNA‐seq analysis combined with reverse target prediction based on protein structure and docking simulations to elucidate the molecular mechanism underlying KMP effects on mitochondrial function. These analyses revealed that key mitochondrial‐related pathways, including the electron transport chain, oxidative phosphorylation, hypoxia response, and apoptosis, were aberrantly activated under simulated microgravity. At the same time, KMP treatment largely reversed these abnormal enrichment patterns. Moreover, by intersecting the predicted KMP‐binding targets with differentially expressed genes identified from RNA‐seq data, we identified four candidate proteins that are both differentially expressed under KMP treatment and have the potential to interact directly with KMP. Among these was NADPH oxidase 4 (NOX4), which demonstrated a high predicted binding affinity with KMP.

NOX4 protein predominantly localizes around mitochondria in chondrocytes and is closely associated with mitochondrial ROS production, extracellular matrix degradation, and cartilage degradation [55, 56]. In OA, NOX4 upregulation has been demonstrated to accelerate cartilage degradation: Renaudin et al. showed that NOX4 deficiency in a DMM‐induced OA mouse model restored cartilage homeostasis by suppressing oxidative stress and inflammation and enhanced anabolic markers such as aggrecan while reducing MMP‐13 and collagen I [57]. Beyond cartilage, studies in cardiac and vascular tissues reveal a broader role for NOX4 in aging, as its expression increases with age and promotes mitochondrial ROS accumulation, dysfunction, and tissue remodeling [58, 59]. Within this mechanistic context, our findings suggest that KMP may directly bind and inhibit NOX4, attenuating mitochondrial ROS and preserving chondrocyte phenotype under simulated microgravity. Collectively, these data highlight NOX4 as a pivotal mediator of OA‐related cartilage degradation and suggest that targeting NOX4 provides a mechanistic basis for KMP's protective effects.

Consistent with this interpretation, we observed that NOX4 expression was reduced in the joint, accompanied by a significant increase in mitochondrial number with KMP treatment, further supporting the conclusion that KMP promotes mitochondrial homeostasis under microgravity conditions. These molecular and structural improvements provide mechanistic evidence for KMP's protective role in cartilage. In summary, our study demonstrates that spaceflight‐simulated microgravity induces detrimental effects on knee articular cartilage, whereas KMP effectively counteracts these changes and preserves cartilage integrity.

It is important to note that this study can be expanded in the future. First, we identified that NOX4 is a critical factor mediating microgravity‐relevant cartilage degradation and tested its roles using cell culture. In the future, its function can be further validated in vivo using animal models. Second, the in vivo studies that we conducted are relatively short (< 7 weeks). Although we observed loss of cartilage matrix, an early sign of OA, we haven't observed structural damage. Therefore, it is still not clear if long‐term spaceflight will directly induce the onset of OA or still needs the involvement of other OA risk factors, such as trauma. Third, pooled cells were used in this study, which masked differences among cells from donors of different ages or disease conditions. At last, the therapeutic effects of KMP were validated only in ground‐based simulated microgravity models; further validation using animals subjected to actual spaceflight conditions is still needed.

## 4 Conclusion

In this study, we demonstrated that the microgravity environment associated with spaceflight induces cartilage loss in the knee joint. Mechanistically, microgravity led to increased mitochondrial ROS production and reduced ATP synthesis in chondrocytes and associated increases in the expression of inflammatory and cellular senescence markers. Kaempferol (KMP) exerted a protective effect by targeting NOX4, suppressing its activity and expression, thereby reducing mitochondrial ROS accumulation and preserving mitochondrial homeostasis. Through this mechanism, KMP effectively protected articular cartilage from microgravity‐induced damage in mice. These findings provide an experimental basis for future spaceflight‐related countermeasures and potential clinical applications.

## 5 Experimental Section

### 5.1 Mice

Upon arrival at the Brookhaven National Laboratory (BNL), the mice were quarantined for two weeks and acclimated to a 12:12 h light:dark cycle with controlled temperature and humidity. Following this, they underwent three days of cage acclimation, with 2 mice per cage. Mice were provided with food and water ad libitum.

Kaempferol (OPC‐KMP) treatment began 4 weeks prior to irradiation, with mice receiving 100 mg/kg/day (5 mg/25 g) via oral gavage. The compound was prepared in a 0.2 mL suspension of 0.5% carboxymethyl cellulose (CMC) at a concentration of 25 mg/mL. Sham‐treated mice received CMC alone using the same administration protocol. After 2 weeks of treatment, mice were randomly assigned to either a normally loaded (NL) or hindlimb unloaded (HU) condition for 2 more weeks. HU was performed using a modified Morey–Holton method adapted for social housing, where mice were suspended by the tail at approximately 30° head‐down tilt, allowing forelimb locomotion while preventing hindlimb weight‐bearing.

After the 4‐week treatment period, the mice were transported to NASA's Space Radiation Laboratory (NSRL) for irradiation. A simplified GCR simulation was performed, delivering a total dose of 0.5 Gy, approximating the radiation astronauts would experience during a Mars round‐trip mission. Sham controls underwent the same handling procedures but were not irradiated. To replicate GCR conditions, mice were positioned in the plateau region of the Bragg curve and irradiated at room temperature for 25 min. The beam consisted of a mixture of high and low LET ions, with 15% high LET and 85% low LET ions, specifically: protons at 1000 and 250 MeV, ^28^Si at 600 MeV/n, ^4^He at 250 MeV/n, ^16^O at 350 MeV/n, and ^56^Fe at 600 MeV/n. The NSRL physics team performed all dosimetry to ensure accuracy.

Following irradiation, the OPC‐KMP treatment continued for an additional week. At the end of the experiment, mice were sacrificed via CO_2_ overdose followed by cervical dislocation, and blood and tissues were collected for analysis. Blood was drawn from the abdominal inferior vena cava (IVC) into EDTA‐coated tubes (0.5 M). Plasma was separated by centrifugation at 1700 × *g* for 15 min and stored at −80°C. A 100 µL cellular fraction was frozen for RNA analysis, and the remaining fraction underwent lysis with RBC lysis buffer (Thermo Fisher). Organs were flash‐frozen or fixed in 4% paraformaldehyde; for example, the heart was bisected vertically, with the left half flash‐frozen and the right half fixed. All tissues were stored at either −80°C or 4°C. Body weights were monitored on days −3, 0, 7, 14, 28, and the day of sacrifice, and all organs were weighed at the time of dissection.

For the spaceflight experiment, 11‐week‐old female C57BL/6J mice were launched to the International Space Station (ISS), where they were housed under microgravity conditions for 60 days (Flight mice). An equal number of mice were hosted on Earth, in a vivarium that simulated the ISS housing conditions (with the exception of the lack of gravity) (Earth mice). Both Flight and Earth mice are part of a separate study sponsored by CASIS/ISS National Laboratory (RR‐25) and, as such, were subjected to the surgical creation of a calvarial critical‐sized bone defect. Being the untreated control mice of the RR‐25 mission, none of them received treatments to induce bone regeneration within the created defect. After 60 days in space or on Earth, mice were euthanized, and knee joint tissues were harvested for comparative analyses.

### 5.2 Histology

Knee joints were dissected, fixed in 4% PFA at 4°C for 24 h, decalcified in Immunocal solution (StatLab, McKinney, TX) for 2 weeks (solution changes every 3 days), dehydrated, embedded in paraffin, and sectioned at 6 µm. Safranin O/Fast Green staining was performed using standard methods.

### 5.3 Modified OARSI Scoring

Histological evaluation of articular cartilage degeneration was performed using a modified OARSI histopathology scoring system based on the recommendations of the OARSI initiative (Glasson et al.) [60]. Knee joint samples were processed into serial sagittal sections and stained with Safranin O–Fast Green to assess cartilage structure and proteoglycan content. Given the sagittal sectioning approach, analysis was focused on the medial compartment of the knee joint, which represents the primary weight‐bearing region and is most susceptible to osteoarthritic changes in the DMM model. For each joint, representative sections from similar locations of the medial compartment were selected for evaluation.

Cartilage degeneration was graded on a scale of 0–6 according to a modified OARSI scoring system, primarily based on the extent (surface width) and depth of cartilage lesions. The medial femoral condyle and medial tibial plateau were scored independently, and the average of these two scores was calculated as the final OARSI score for each sample. All histological scoring was performed by two independent observers in a blinded manner to minimize bias.

### 5.4 Immunohistochemistry

Paraffin sections were rehydrated, blocked with 10% horse serum (Vector Labs, S‐2000‐20) in PBS for 1 h, and incubated overnight at 4°C with primary antibodies (Table S1). Slides were then incubated sequentially with biotinylated secondary antibodies (1 h), HRP‐conjugated streptavidin (45 min; Vectastain Elite ABC kit, Vector Labs, PK‐6101), and developed with NovaRed substrate. Sections were counterstained with hematoxylin, dehydrated, and mounted.

### 5.5 hMSC Isolation and Culture

Primary hMSCs were isolated as previously described [61]. Human mesenchymal stem cells (hMSCs) were isolated from femoral heads obtained from de‐identified donors under Institutional Review Board (IRB) approval from the University of Pittsburgh and the University of Washington. Isolation was performed as previously described. Briefly, bone marrow aspirates were collected from femoral heads and filtered through a 70‐µm cell strainer to remove debris. The mononuclear cell fraction was separated by density‐gradient centrifugation and plated in growth medium (DMEM; Gibco) supplemented with 10% (*v/v*) fetal bovine serum (FBS; Gibco), 1% (*v/v*) antibiotic‐antimycotic (Gibco), and 1.5 ng/ml fibroblast growth factor‐2 (FGF‐2; PeproTech). Cells were maintained at 37°C in a humidified 5% CO_2_ atmosphere, and medium was changed twice weekly. When cultures reached 70%–80% confluence, cells were detached with 0.25% (*w/v*) trypsin‐EDTA (Thermo Fisher Scientific) and passaged at a 1:3 ratio. To obtain sufficient cell numbers to complete this study, MSCs were pooled from 3 male and 3 female donors, aged 24–79 years.

### 5.6 Chondrocyte Isolation

Healthy human knee cartilage was obtained from arthritis‐free decedents (Oversight of Research and Clinical Training Involving Decedents approval, University of Pittsburgh). Chondrocytes were isolated from articular cartilage as previously described [62], enzymatically released, and plated in high‐glucose DMEM (Gibco/Thermo Fisher Scientific) supplemented with 10% FBS (Life Technologies) and 1% antibiotic–antimycotic (Life Technologies). Cells were maintained at 37°C, 5% CO_2_ with medium changes twice weekly. At 80%–90% confluence, cells were detached with 0.25% trypsin–EDTA (Thermo Fisher Scientific) and passaged. To limit dedifferentiation and ensure sufficient numbers while reducing donor variability, P1 healthy chondrocytes pooled from 16 donors (1:1 male:female; ages 15–74 years) were used for experiments.

### 5.7 Hydrogel Synthesis and Cell Encapsulation

Methacrylated hyaluronic acid (HA; Advanced BioMatrix, CA, USA) was dissolved in PBS at 2% (*w/v*) with 0.15% LAP photoinitiator. Human MSCs were suspended at 20 × 10^6^ cells/mL and cast in silicone molds (6 mm diameter, 2 mm height). Constructs were crosslinked under 395 nm light for 2 min. After 28 days in chondrogenic medium (DMEM, 0.1 µM dexamethasone, 10 µg/mL ITS (Insulin–Transferrin–Selenium), 40 µg/mL L‐proline, 50 µg/mL ascorbate‐2‐phosphate, 10 ng/mL TGFβ3 (Transforming Growth Factor Beta 3)), hydrogels were used for loading and rotary culture.

### 5.8 Rotary Cell Culture System (Microgravity Simulation)

hMSC‐derived cartilage constructs were cultured in a Rotary Cell Culture System (RCCS; Synthecon Inc., Houston, TX, USA) to simulate a microgravity environment. Each bioreactor vessel was filled with chondrogenic medium to eliminate air bubbles and minimize shear stress. The system was operated at a rotation speed of 32.5 revolutions per minute (RPM) inside a standard humidified cell culture incubator (37°C, 5% CO_2_). Under these conditions, the constructs remained in continuous free fall, providing a dynamic, low‐shear environment that promotes nutrient exchange and three‐dimensional tissue formation. Gels were maintained in chondrogenic differentiation medium consisting of high‐glucose DMEM supplemented with 1 × ITS, 0.5 ng/mL TGF‐β3, 50 µg/mL ascorbic acid, and 1% penicillin–streptomycin. Medium was refreshed every two to three days. Samples were harvested on days 3, 7, and 14 for subsequent analyses.

### 5.9 Establishment of an OA‐Like Cartilage Model Using P9 hMSC‐Derived Constructs in a Rotary Cell Culture System

To further investigate OA‐like changes under simulated microgravity conditions, cartilage constructs, generated from passage 9 human mesenchymal stem cells (P9 hMSCs) using the same chondrogenic culture described above [27], were cultured in a Rotary Cell Culture System (RCCS; Synthecon Inc., Houston, TX, USA). The details of the experiments were prepared as described in Section Rotary Cell Culture System (Microgravity Simulation). Samples were harvested on day 7 for subsequent analysis.

### 5.10 Bulk RNA‐seq

hMSC‐derived cartilage constructs from static, rotary, or rotary + Kaempferol (KMP) hydrogels were lysed in QIAzol reagent (Qiagen). RNA was quantified (Qubit RNA BR Assay Kit, Thermo Fisher) and integrity assessed (Fragment Analyzer, Agilent). Libraries were prepared with the KAPA mRNA HyperPrep Kit (Roche) using 500 ng RNA per sample, validated, pooled, and sequenced (Illumina NovaSeq6000, 50 million 100‐bp paired‐end reads/sample).

Reads were quality‐checked (FastQC), trimmed (Trimmomatic), aligned to mm10 (STAR), and quantified at the gene level. Differential expression was analyzed with DESeq2 (adjusted *p* ≤ 0.05, fold change ≥ 1.5). GO and KEGG enrichment, PCA, and GSEA were performed. Pathways with FDR < 0.05 were considered significant.

### 5.11 Real‐time qPCR

RNA was isolated with QIAzol and QIAwave RNA Mini Kit (Qiagen), reverse transcribed with SuperScript IV Vilo Master Mix (Thermo Fisher), and amplified with PowerUp SYBR Green Master Mix (Thermo Fisher). Cycling: 95°C 15 s, 60°C 60 s (40 cycles). Primer sequences are listed in Table S2. Relative expression was calculated by ΔΔCT against sham controls. Abbreviations of all genes used in this study are listed in Table S3.

### 5.12 Western Blot

Proteins were extracted with RIPA buffer (Sigma–Aldrich) containing Halt protease/phosphatase inhibitors. Samples were denatured at 99°C for 7 min, resolved by SDS‐PAGE (4%–12% Bis–Tris gels, Invitrogen), and transferred to membranes. Membranes were blocked (Bio‐Rad everyBlot buffer), probed overnight at 4°C with primary antibodies, followed by HRP‐conjugated secondary antibodies (Abcam), and visualized with SuperSignal West Dura (Thermo Fisher) on ChemiDoc Touch (Bio‐Rad). Bands were quantified with ImageJ.

### 5.13 Molecular Docking

Docking was performed using AutoDock Vina (v1.2.3) with protein structures (UniProt ID Q9NPH5). Ligands and proteins were prepared in AutoDockTools (v1.5.7). Binding poses with the lowest binding energy were visualized with PyMOL (v2.3.0) and LigPlot+ (v2.2.8).

### 5.14 CCK8 Cytotoxicity Assay

Human primary chondrocytes (1 × 10^4^ cells/well) were seeded in 96‐well plates, treated with graded Kaempferol concentrations, and viability was measured at days 0, 1, 3, and 7 using CCK8 (Abcam, ab228554).

### 5.15 SiRNA Transfection

Human primary chondrocytes (∼70% confluence) were transfected with siRNA against NOX4 using Lipofectamine RNAiMAX (Thermo Fisher). Medium was replaced after 48 h, and cells were harvested 72 h post‐transfection.

### 5.16 Dcfda ROS Assay

hMSC‐derived cartilage constructs were incubated with ROS‐sensitive dye (1–10 µM) for 5–60 min, washed, and allowed to recover. Baseline and stimulated fluorescence were measured with appropriate negative (unstained, dye‐only) and positive controls (H_2_O_2_, TBHP).

### 5.17 Mitochondrial Stress Test

Human primary chondrocytes were seeded in XFe96 Spheroid plates. Seahorse cartridges were hydrated overnight in XF calibrant. On assay day, inhibitors were loaded: Oligomycin (2 µM), FCCP (1 µM), Rotenone/Antimycin A (0.5 µM). Measurements were performed on the Seahorse XFe96 Analyzer (Agilent).

### 5.18 Surface Plasmon Resonance

NOX4 was immobilized on CM5 chips using EDC/NHS activation. Kaempferol (1–100 µM) was injected at 20 µL/min for 100 s association and 180 s dissociation. Sensorgrams were analyzed after regeneration between cycles.

### 5.19 Kaempferol (OPC‐KMP) Treatment

Kaempferol (OPC‐KMP) was administered via oral gavage at 100 mg/kg/day (5 mg/25 g mouse) for 4 weeks before irradiation. The compound was suspended in 0.2 mL of 0.5% carboxymethyl cellulose (CMC; 25 mg/mL). Sham‐treated mice received CMC alone. Treatment continued for 1 week after irradiation.

### 5.20 Statistical Analysis

Statistical analyses were performed using GraphPad Prism 9 (GraphPad Software) and SPSS v25 (IBM). Data are presented as mean ± standard deviation (SD). For two‐group comparisons, unpaired two‐tailed Student's t‐tests were used. For comparisons among three or more groups, one‐way ANOVA followed by Holm–Šídák's multiple comparisons test was performed. *p* < 0.05 was considered statistically significant.

## Author Contributions

Conceptualization, A.B., H.L.; methodology and samples, Y.Y., M.Z., R.C., M.F.A., A.G., S.D., R.S., J.C.S., A.H.Z., N.G.A., A.J.K., E.A.B., G.I.; investigation, Y.Y., R.X., P.G.A., H.L., A.B.; ‘omic data analyses, R.X., J.L., A.B, and S.L.; writing – original draft, Y.Y., H.L., and A.B.; writing – review & editing, Y.Y., H.L., A.B., A.Z.O., W.X., D.C.W., C.E.M, G. I. All authors read and approved the paper.

## Conflicts of Interest

The authors declare no conflicts of interest.

## Supporting information




**Supporting File**: advs76477‐sup‐0001‐SuppMat.pdf.

## Data Availability

The authors declare that the relevant data supporting the findings of this study are available within the paper and its supplementary files. The detailed sequencing data have been uploaded to the GEO database with accession ID GSE310264 (https://www.ncbi.nlm.nih.gov/geo/query/acc.cgi?acc=GSE310264). During the review process, please enter the token knqpugaklpcltop into the box to access the data.
