# Supplementary material for: Kaempferol Attenuates Spaceflight‐Associated Knee Cartilage Degradation by Targeting NOX4‐Mediated Mitochondrial Dysfunction
Source: Adv Sci (Weinh). 2026 Jul 9:e76477. Online ahead of print. doi: 10.1002/advs.76477 (PMC13348333; doi:10.1002/advs.76477)
Supplement: Supplementary file 1 — Supporting File: advs76477‐sup‐0001‐SuppMat.pdf. [file ADVS-9999-e76477-s001.pdf]

Supplementary Materials

Yuesong Yin[1#], Ruiling Xu[1#], Meagan J. Makarczyk[1,2], Jia-Jun Liu[3,4], Silvia Liu[3,4], Matthew Z Shi[1], Roberta Di Carlo[5,6], Michael F. Almeida[7], Alex Garris[7], Sarah Day[7], Rebekah Sanchez-Hodge[7], Jonathan C. Schisler[7], Aleeza H. Zilberman[8], Noah G. Allen[8], Angela J. Kubik[8], Elizabeth A. Blaber[8], Arnold Z. Olali[9], Wanqing Xie[9], Douglas C Wallace[9,10], Christopher E. Mason[11], Peter G. Alexander[1, 12], Giuseppe Intini[5,6,13,14], Afshin Beheshti[14,15,16,17,18*], Hang Lin[1,2,12,15 *]

[1]Department of Orthopaedic Surgery, University of Pittsburgh School of Medicine, Pittsburgh, PA, USA

[2]Department of Bioengineering, University of Pittsburgh Swanson School of Engineering, Pittsburgh, PA, USA

[3]Department of Pharmacology and Chemical Biology, University of Pittsburgh School of Medicine, Pittsburgh, PA, USA

[4]Organ Pathobiology and Therapeutics Institute, University of Pittsburgh School of Medicine, Pittsburgh, PA, USA

[5]Department of Periodontics and Preventive Dentistry, University of Pittsburgh School of Dental Medicine, Pittsburgh, PA, USA

[6]Center for Craniofacial Regeneration, University of Pittsburgh School of Dental Medicine, Pittsburgh, PA, USA

[7]McAllister Heart Institute and Department of Pharmacology, The University of North Carolina at Chapel Hill, Chapel Hill, NC, USA

[8]Shirley Ann Jackson, Ph.D. Center for Biotechnology and Interdisciplinary Studies and Department of Biomedical Engineering, Rensselaer Polytechnic Institute, Troy, NY, USA

[9]Center for Mitochondrial and Epigenomic Medicine, The Children's Hospital of Philadelphia, Philadelphia, PA, USA

[10]The Division of Human Genetics, The Department of Pediatrics, The Perelman School of Medicine, University of Pennsylvania, Philadelphia, PA, USA

[11]Department of Physiology and Biophysics, Weill Cornell Medicine, New York, NY, USA

[12]Orland Bethel Family Musculoskeletal Research Center, University of Pittsburgh School of Medicine, Pittsburgh, PA, USA

[13]University of Pittsburgh UPMC Hillman Cancer Center, Pittsburgh, PA, USA

[14]Center for Space Biomedicine, University of Pittsburgh School of Medicine, Pittsburgh, PA, USA

[15]McGowan Institute for Regenerative Medicine, University of Pittsburgh School of Medicine, Pittsburgh, PA, USA

[16]Department of Surgery, University of Pittsburgh School of Medicine, Pittsburgh, PA, USA

[17]Department of Computational and Systems Biology, University of Pittsburgh School of Medicine, Pittsburgh, PA, USA

[18]Stanley Center for Psychiatric Research, Broad Institute of MIT and Harvard, Cambridge, MA, USA

#These two authors contribute equally to this work.

Figure S1.

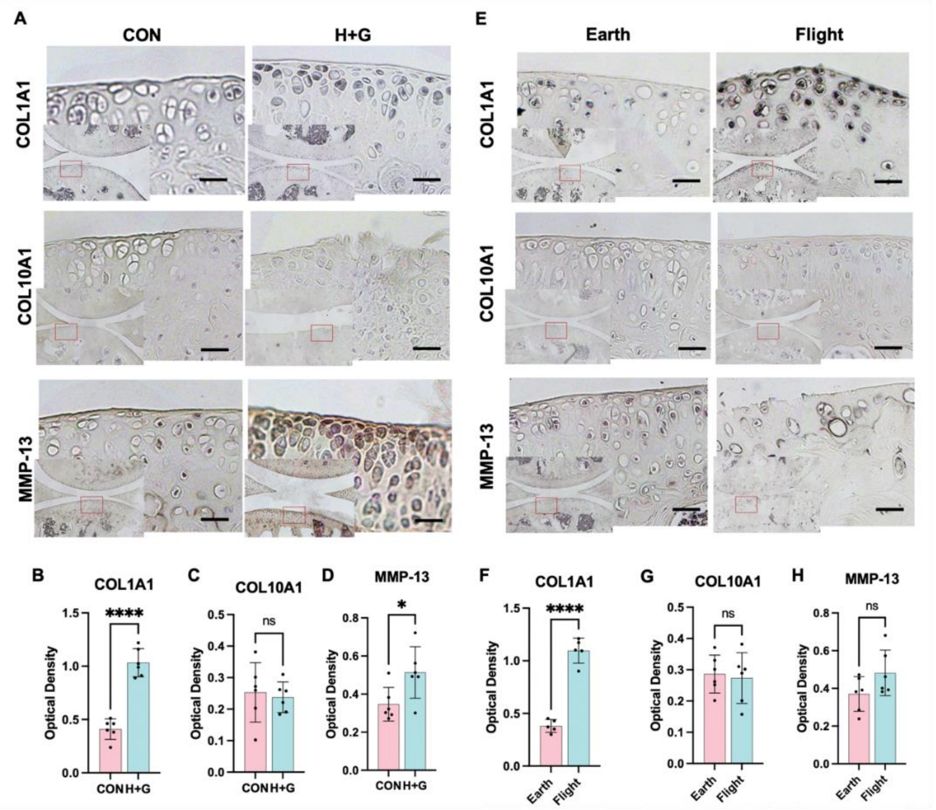

Figure S1. (A, E) Representative immunohistochemical staining of collagen type I alpha 1 (COL1A1), collagen type X alpha 1 (COL10A1), and matrix metalloproteinase-13 (MMP-13) in knee joint sections from mice subjected to (A) simulated microgravity (CON vs. H+G) and (E) spaceflight (Earth vs. Flight). Red boxes in lower-magnification images indicate the locations of higher-magnification images. (B–D) Semi-quantitative analysis of (B) COL1A1, (C) COL10A1, and (D) MMP-13 staining in CON and H+G groups. (F–H) Semi-quantitative analysis of (F) COL1A1, (G) COL10A1, and (H) MMP-13 staining in Earth and Flight groups. Staining intensity was quantified as optical density. Scale bars: 25 μm. n = 5–6 mice per group. Data are presented as mean ± SD. ns, not significant; *$p < 0.05$; ****$p < 0.0001$. Statistical significance was determined using an unpaired two-tailed Student's t-test.

Figure S2.

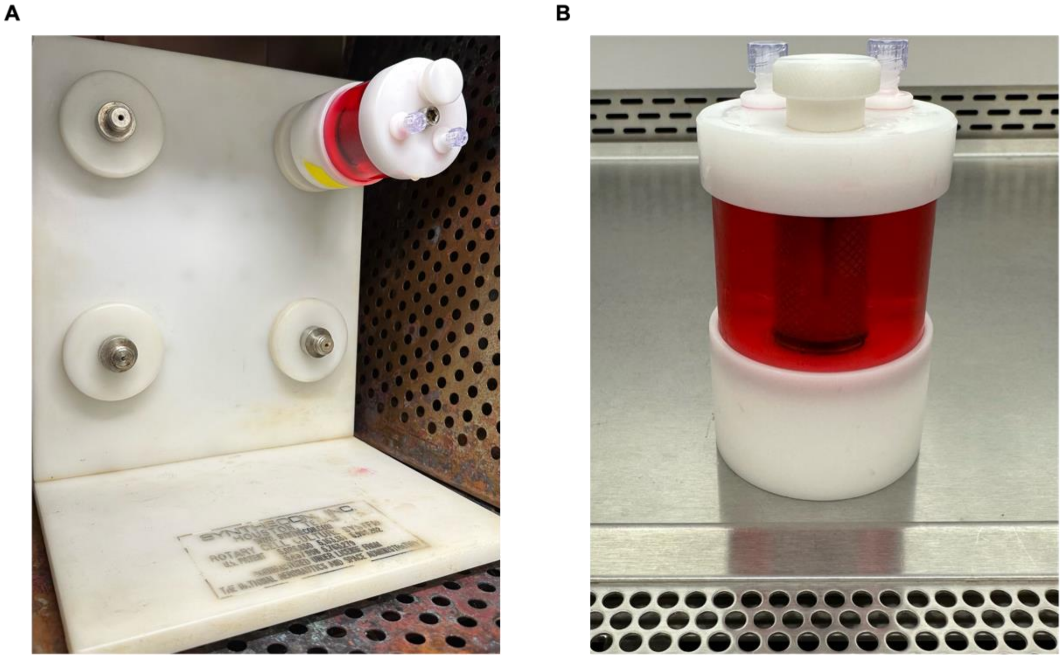

Figure S2. (A) Setup of the Rotary Cell Culture System (RCCS); (B) Photograph of the RCCS unit.

Figure S3.

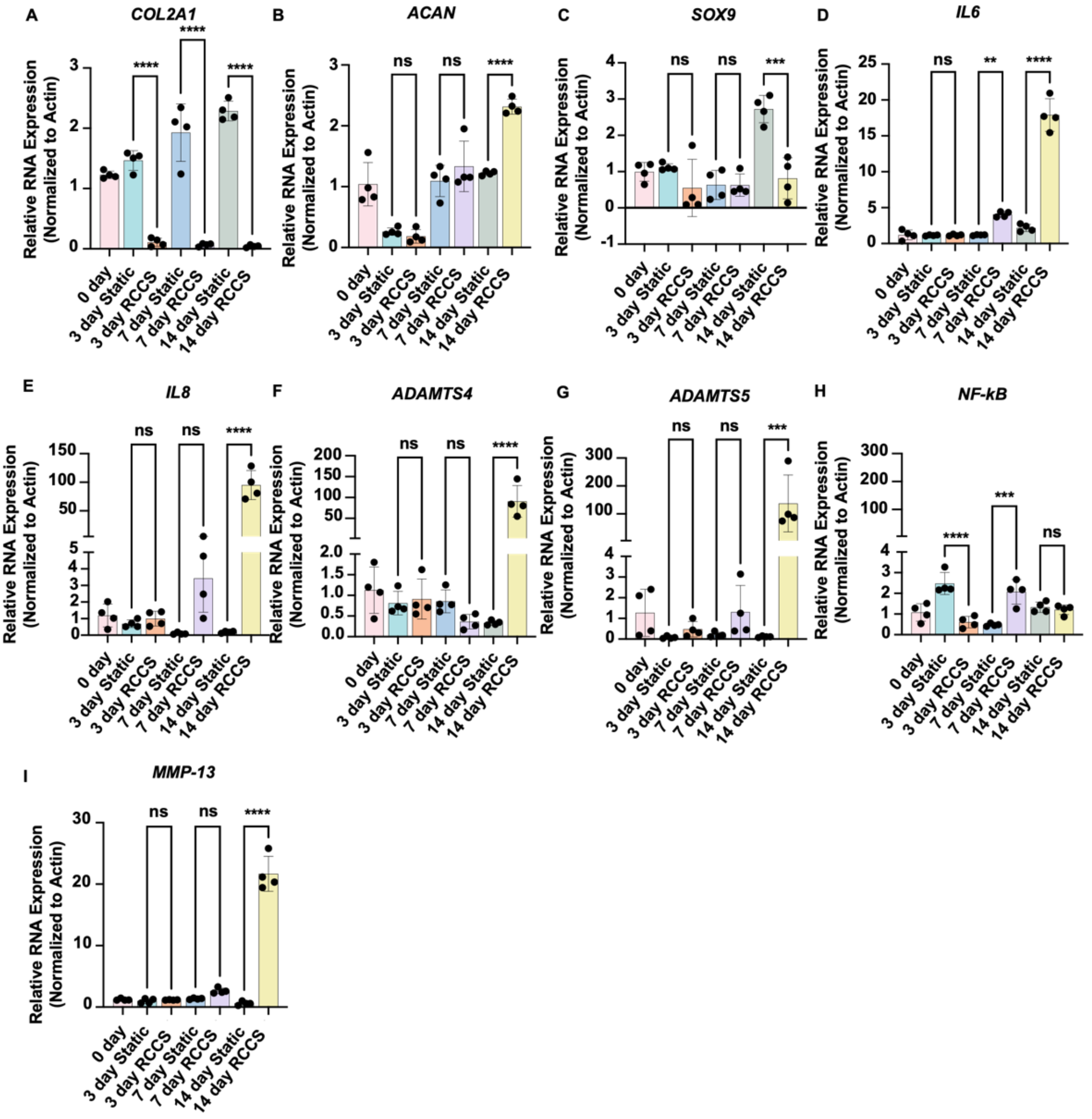

Figure S3. qRT-PCR analysis of chondrocytes cultured under static or RCCS conditions for 3, 7, and 14 days. (A-C) Expression levels of chondrogenic markers. (D, I) Expression levels of pro-inflammatory cytokines and enzymes breaking down cartilage. Data are presented as mean ± SD. n=4. *One-way ANOVA followed by Holm-Šídák's multiple comparisons test was performed*. **, $p < 0.01$; ***, $p < 0.001$, ****, $p < 0.0001$; ns, not significant. Abbreviations: *COL2A1*, collagen type II alpha 1 chain; *ACAN*, aggrecan; *SOX9*, SRY-box transcription factor 9; *IL6*, interleukin 6; *IL8,* interleukin 8; *ADAMTS 4*, ADAM metallopeptidase with thrombospondin type 1 motif 4; *ADAMTS 5*, ADAM metallopeptidase with thrombospondin type 1 motif 5; *NF-kB*, nuclear factor kappa B; *MMP13*, matrix metallopeptidase 13.

Figure S4.

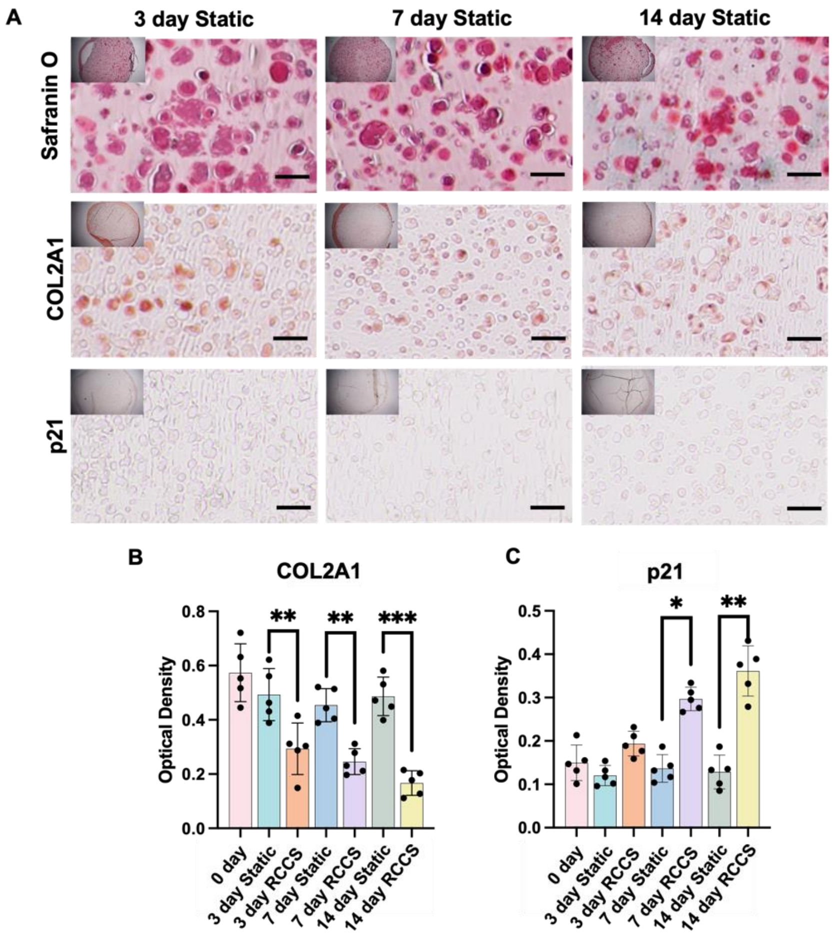

Figure S4. (A) Representative images of Safranin O staining and immunohistochemical staining for COL2A1 and p21 in cartilage constructs cultured under static conditions for 3, 7, and 14 days. Scale bars: 10 µm; insets show low-magnification views of whole constructs. (B, C) Quantification of staining intensity (optical density) for COL2A1 (B), and p21 (C) across all experimental groups, including day 0 baseline, static and rotary culture at 3, 7, and 14 days. Data are presented as mean ± SD (n = 5 per group). *One-way ANOVA followed by Holm-Šídák's multiple comparisons test was performed*. *, $p < 0.05$; **, $p < 0.01$; ***, $p < 0.001$.

Figure S5.

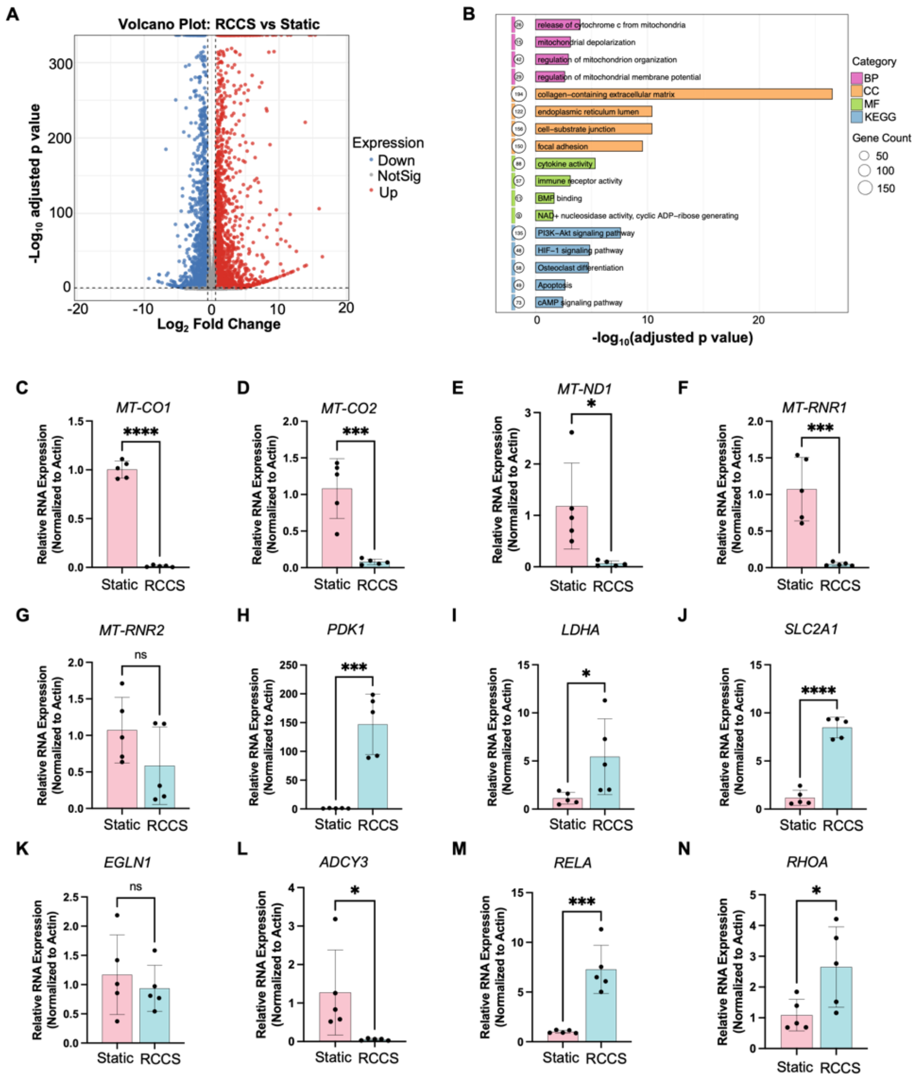

Figure S5. (A) Volcano plot showing differentially expressed genes between RCCS and static groups. Red dots represent upregulated genes, blue dots represent downregulated genes, and gray dots indicate non-significant changes. (B) Bar plot of significantly enriched GO/KEGG pathways ranked by –log10 adjusted p value. Categories include biological process (BP), cellular component (CC), molecular function (MF), and KEGG pathways. (C–G) Relative expression of mitochondrially encoded genes *MT-CO1*, *MT-CO2*, *MT-ND1*, *MT-RNR1*, and *MT-RNR2* under static versus rotary culture conditions. (H–J) Relative expression of glycolysis and metabolic reprogramming genes *PDK1*, *LDHA*, and *SLC2A1*. (K–N) Relative expression of mechanosensitive and inflammation-related genes *EGLN1*, *ADCY3*, *RELA*, and *RHOA*. Statistical significance was determined by unpaired two-tailed t-test. *, $p < 0.05$; **,

$p < 0.01$; ***, $p < 0.001$; ****, $p < 0.0001$; ns, not significant. Abbreviations: *MT-CO1*, mitochondrially encoded cytochrome c oxidase I; *MT-CO2*, mitochondrially encoded cytochrome c oxidase II; *MT-ND1*, mitochondrially encoded NADH dehydrogenase 1; *MT-RNR1*, mitochondrially encoded 12S RNA; *MT-RNR2*, 16S rRNA, mitochondrial; *PDK1*, pyruvate dehydrogenase kinase 1; *LDHA*, lactate dehydrogenase A; *SLC2A1*, solute carrier family 2 member 1; *EGLN1*, egl-9 family hypoxia inducible factor 1; *ADCY3*, adenylate cyclase 3; *RELA*, RELA proto-oncogene, NF-kB subunit; *RHOA*, ras homolog family member A.

Figure S6.

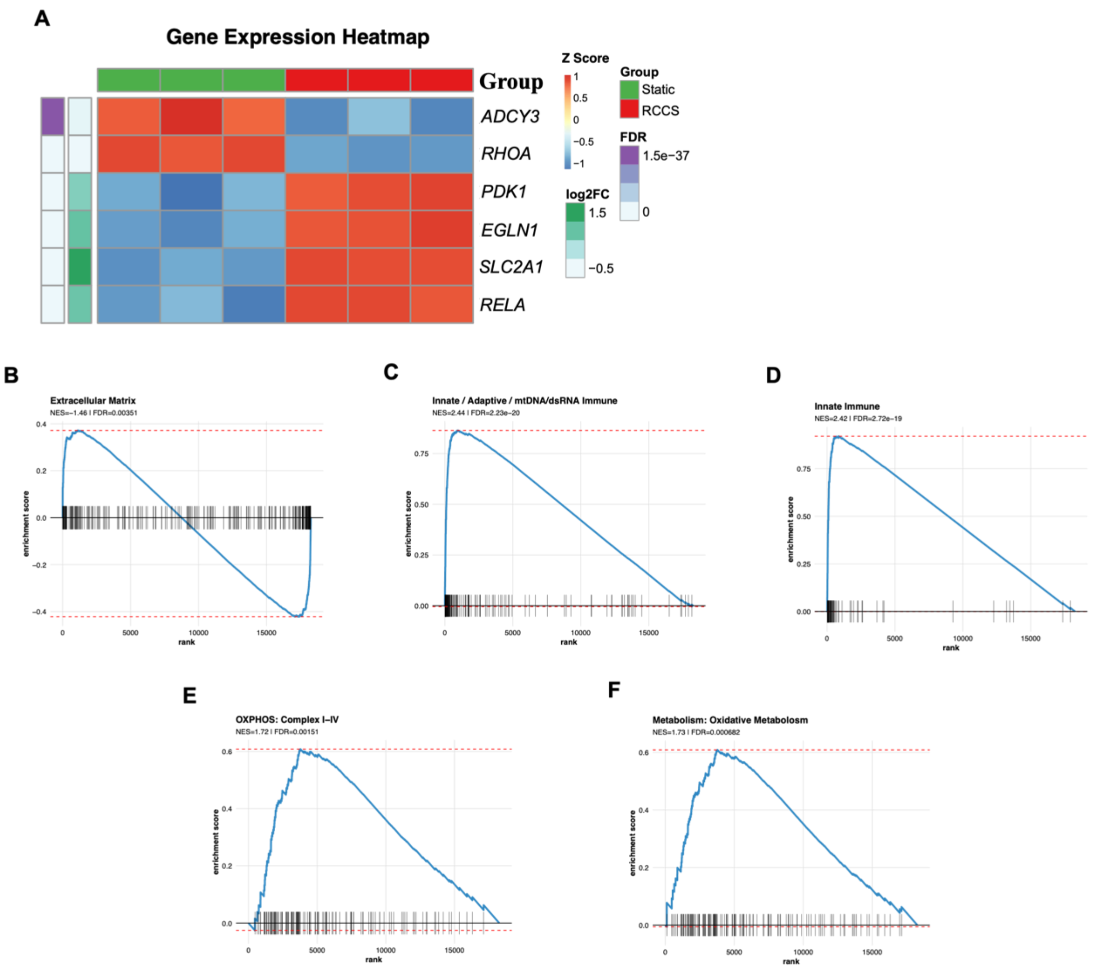

Figure S6. (A) Heatmap showing the expression profiles (Z score) of representative genes (*ADCY3*, *RHOA*, *PDK1*, *EGLN1*, *SLC2A1*, *RELA*) from RNA-Seq, showing a similar trend to the results from qRT-PCR. (B)Extracellular Matrix, (C) Innate/adaptive immune and mtDNA/sRNA immune responses, (D) Innate Immune, (E) Oxidative phosphorylation (OXPHOS): complexes I–IV, and (F) Oxidative metabolism pathways (F) in RCCS versus static culture.

Figure S7.

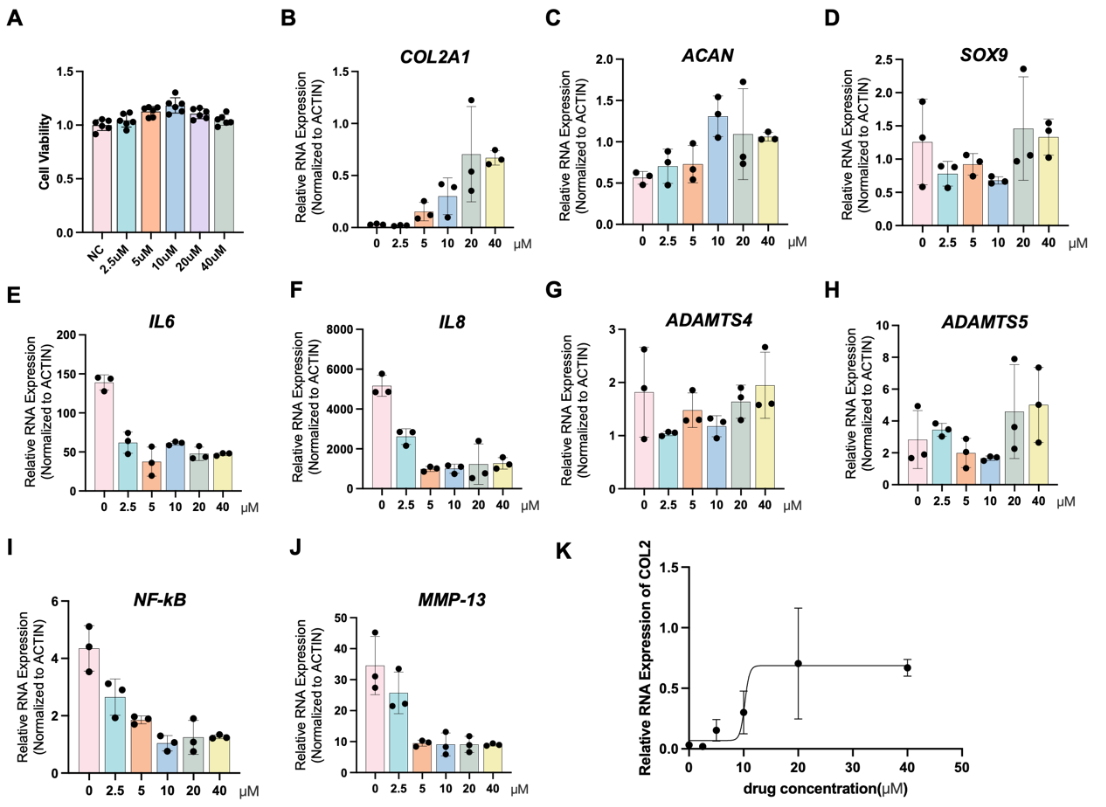

Figure S7. (A) Cell viability after KM treatment (2.5–40 μM) was measured by CCK8 assay. (n = 6 per group) (B–J) qRT-PCR to examine gene expression in chondrocytes treated with KMP from 0 to 40 μM (n=3 per group). (K) Dose–response curve of COL2 expression upon KMP treatment. Data are shown as mean ± SD. *One-way ANOVA followed by Holm-Šídák's multiple comparisons test was performed*. *, $p < 0.05$; **, $p < 0.01$; ***, $p < 0.001$; ****, $p < 0.0001$; ns, not significant.

Figure S8.

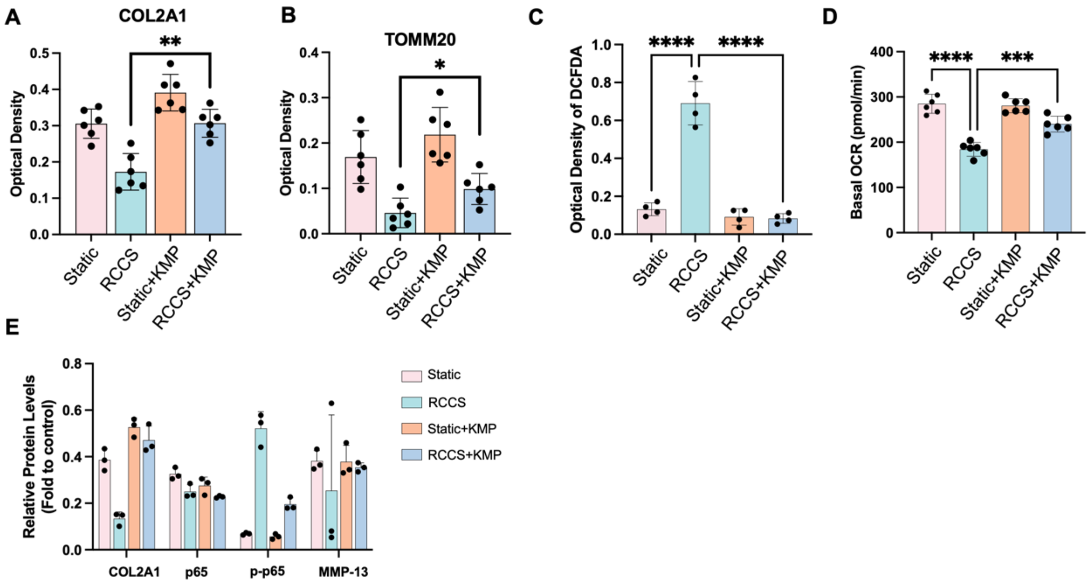

Figure S8. (A and B) Immunohistochemical quantification of COL2 (A) and mitochondrial marker TOMM20 (B) in constructs cultured under Static or RCCS conditions with or without KMP treatment (Static, RCCS, Static+KMP, and RCCS+KMP groups); n=6. (C) Quantification of DCFDA staining; n=4. (D) Basal oxygen consumption rate (OCR) measured by Seahorse XF assay; n=6. (E) Semi-quantification of Western blot analysis of protein levels in different treatment groups; n=3. Data are shown as mean ± SD. *One-way ANOVA followed by Holm-Šídák's multiple comparisons test was performed*. **, $p < 0.01$; ***, $p < 0.001$; ****, $p < 0.0001$; ns, not significant.

Figure S9.

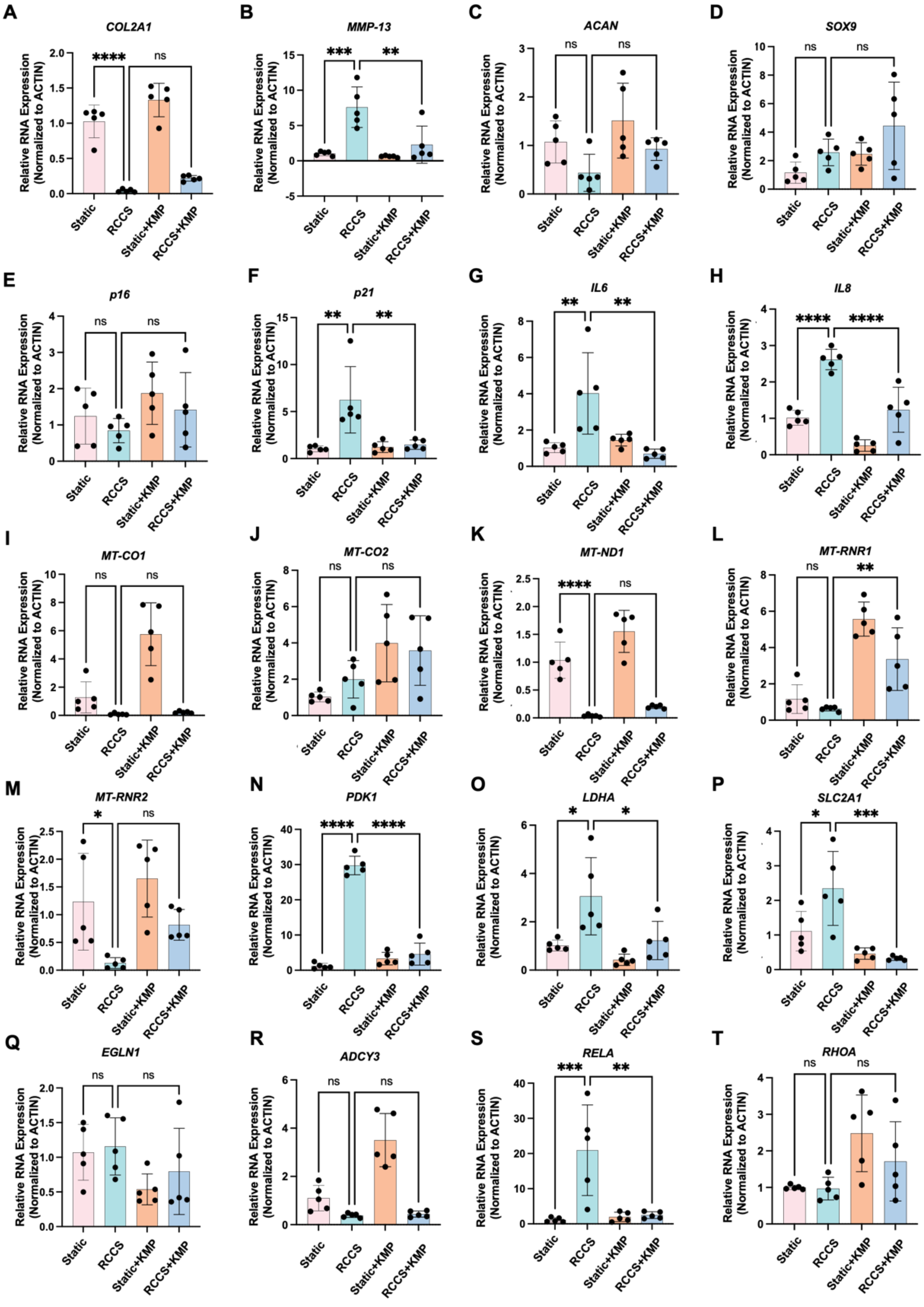

Figure S9. (A-T) qRT-PCR to examine the gene expression in chondrocytes under static or rotary (RCCS) conditions, with or without kaempferol (KMP, 20 µM) treatment. Data are shown as mean ± SD (n = 5 per group). *One-way ANOVA followed by Holm-Šídák's multiple comparisons test was performed*. *, $p < 0.05$; **, $p < 0.01$; ***, $p < 0.001$; ****, $p < 0.0001$; ns, not significant.

Figure S10

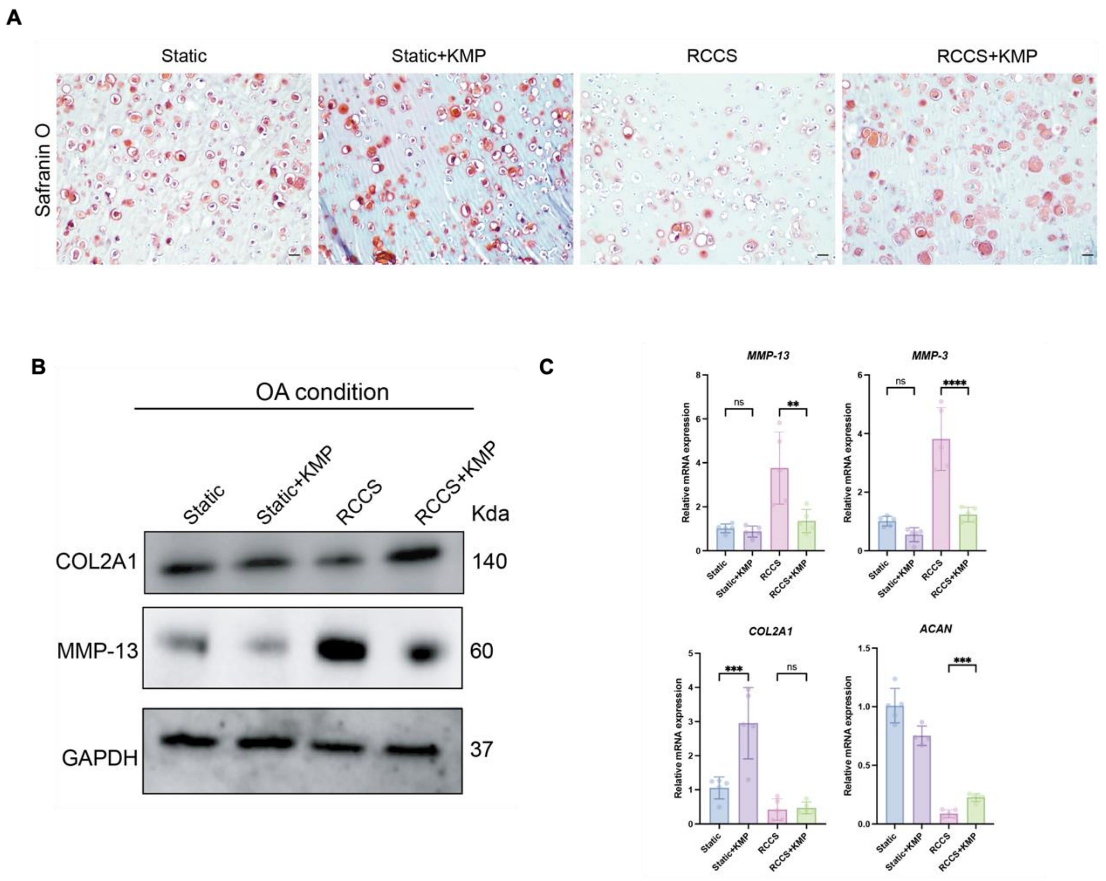

Figure S10. (A) Representative Safranin O staining of OA chondrocytes subjected to static culture, static with KMP treatment (Static+KMP), rotary cell culture system (RCCS), and RCCS with KMP treatment (RCCS+KMP). (B) Western blot analysis of collagen type II alpha 1 (COL2A1) and matrix metalloproteinase-13 (MMP-13) protein expression in OA chondrocytes under the indicated conditions. GAPDH was used as a loading control. (C) Quantitative real-time PCR analysis of MMP-13, MMP-3, COL2A1, and aggrecan (ACAN) mRNA expression in OA chondrocytes across the four experimental groups. Data are presented as mean ± SD from three independent experiments. ns, not significant; **$p < 0.01$; ***$p < 0.001$; ****$p < 0.0001$. *One-way ANOVA followed by Holm-Šídák's multiple comparisons test was performed*.

Figure S11.

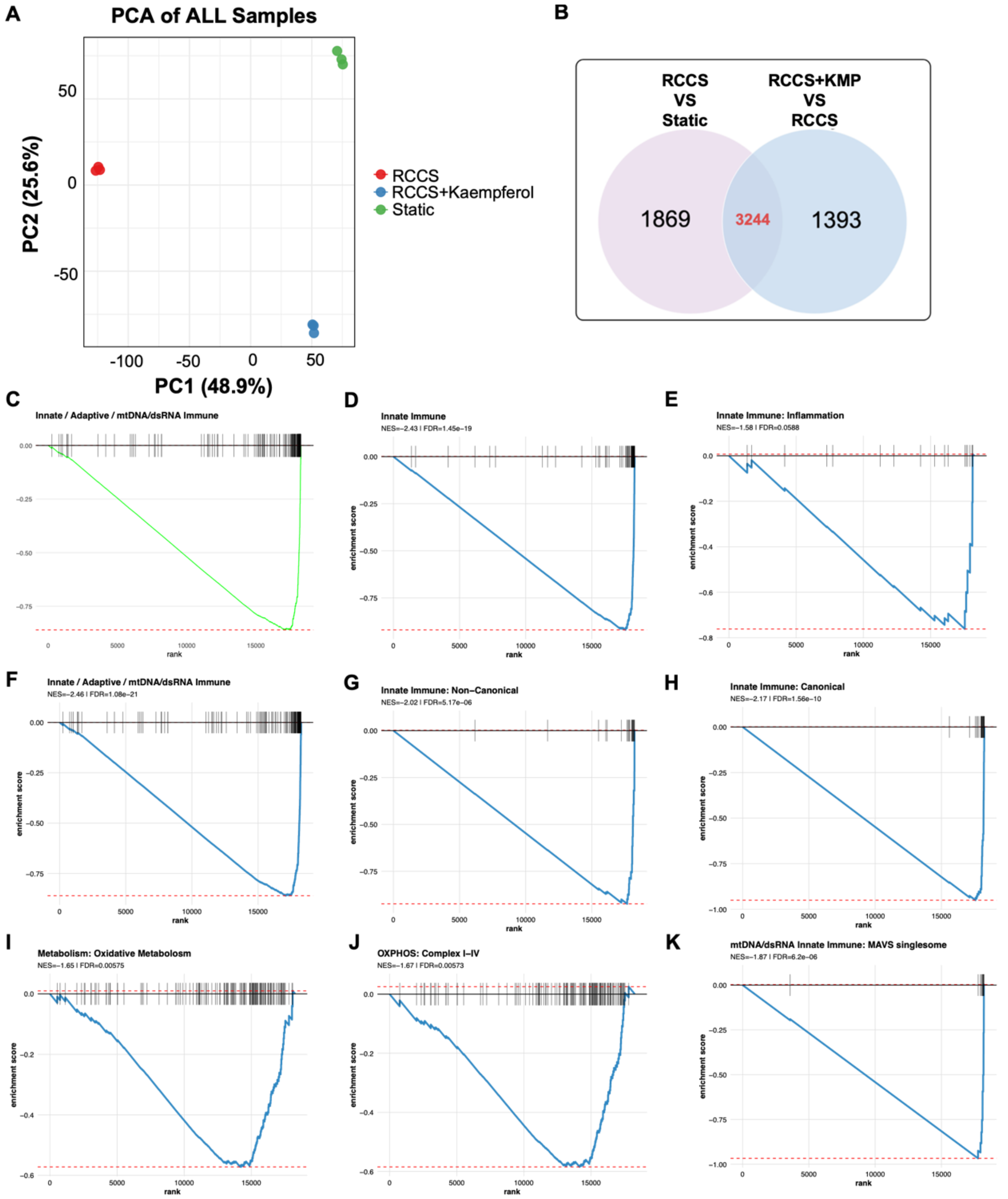

Figure S11. Transcriptomic profiling. (A) PCA plot showing distinct clustering of static, RCCS, and RCCS + kaempferol (KMP) groups. (B) Venn diagram indicating the overlap of differentially expressed genes (DEGs) between rotary vs static and kaempferol vs rotary comparisons. (C–K) Representative GSEA plots showing suppression of innate/adaptive immune and inflammatory pathways (C–H) and restoration of oxidative metabolism and oxidative phosphorylation (I–J) by KMP treatment, as well as reversal of mtDNA/sRNA innate immune activation (K).

Figure S12.

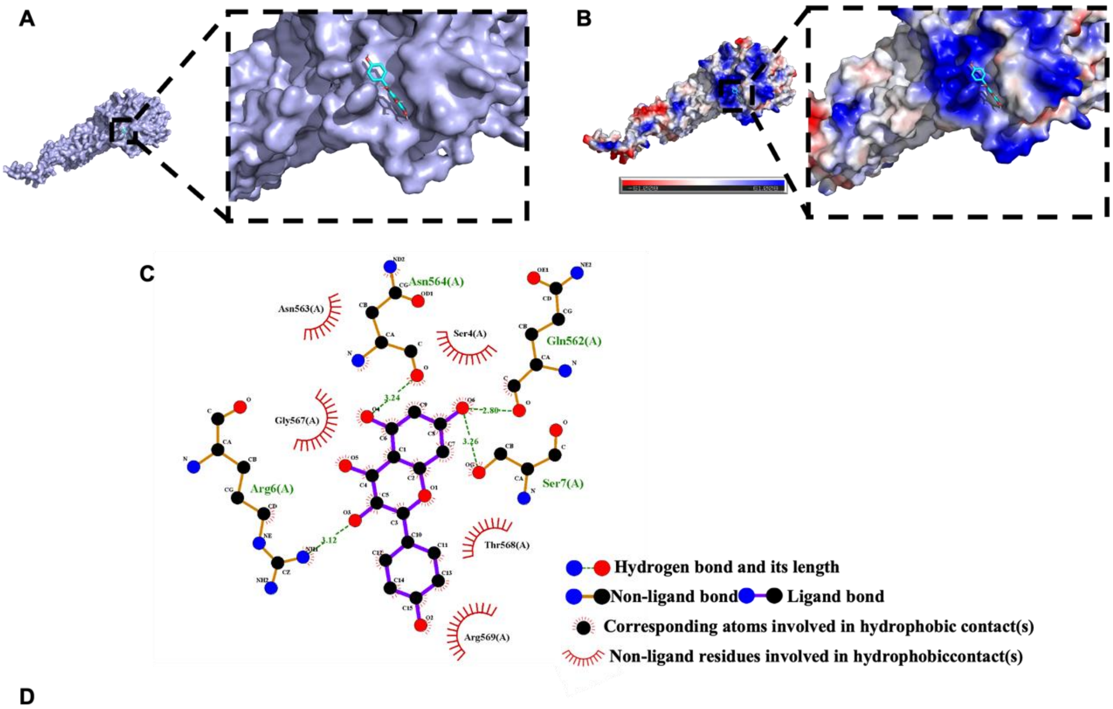

D

mode	Affinity (kcal/mol)	rmsd l.b.	rmsd u.b
1	-7.4	0.000	0.000
2	-6.6	24.770	26.866
3	-6.4	2.662	5.803
4	-6.3	17.004	17.477
5	-6.2	17.227	17.905
6	-6.1	37.157	39.090
7	-6	36.214	39.400
8	-5.9	36.445	38.393
9	-5.9	17.065	20.450

Figure S12. Molecular docking reveals KM binding to NOX4 and its expression is altered by rotary culture. (A) Predicted binding pose of KM to NOX4 protein shown in surface representation. Zoomed-in view highlights the binding pocket. (B) Electrostatic surface potential of NOX4 with KM docked in the active site. Positive (blue), negative (red), and neutral (white) regions are shown. (C) 2D interaction diagram of KM and NOX4. Hydrogen bonds (blue lines), ligand bonds (black), hydrophobic contacts (red arcs), and non-ligand interactions are illustrated. (D) Table showing docking results from AutoDock, listing binding affinities (kcal/mol) and RMSD values across the top nine predicted modes.

Figure S13

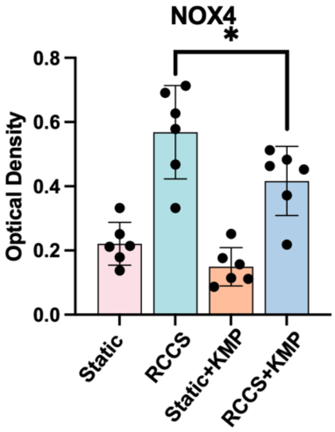

Figure S13. Quantification of NOX4 protein levels by immunostaining across Static, RCCS, Static+KMP, and RCCS+KMP groups. Data are shown as mean ± SD (n = 5). *One-way ANOVA followed by Holm-Šídák's multiple comparisons test was performed*. *, $p < 0.05$.

Figure S14.

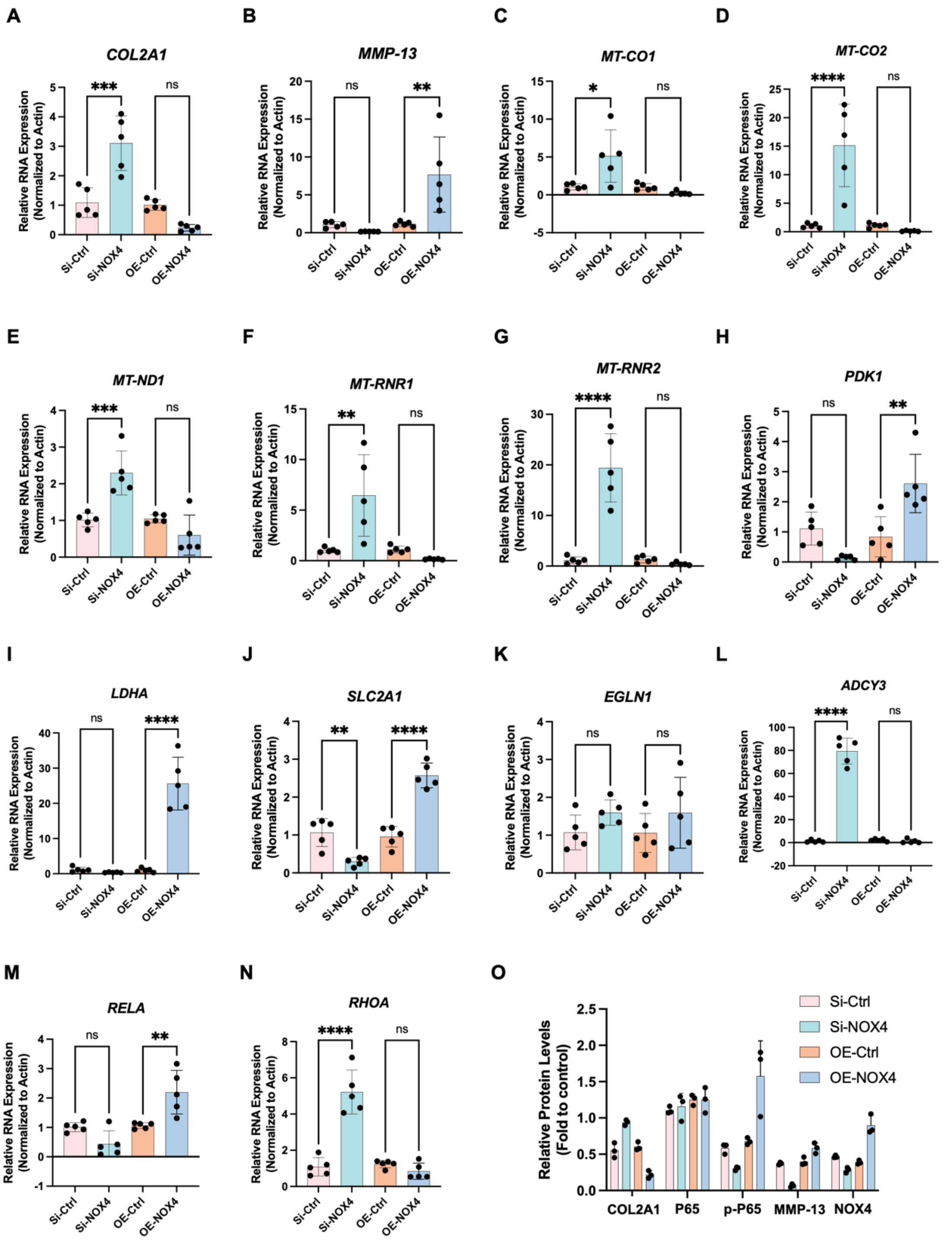

Figure S14. (A-N) qRT-PCR to examine the gene expression in chondrocytes treated with NOX4 siRNA (si-NOX4) or lentiviral vectors carrying the NOX4 gene (OE-NOX4). Scrambled siRNA (si-Ctrl) and lentiviral vectors carrying a control gene (OE-Ctrl) were used as the control, respectively. Data are presented as mean ± SD, n = 5. *One-way ANOVA followed by Holm-Šídák's multiple comparisons test was performed*.

*, $p < 0.05$; **, $p < 0.01$; ***, $p < 0.001$; ****, $p < 0.0001$; ns, not significant. (O) Western blot quantification showing relative protein levels of COL2, P65, p-P65, MMP13, and NOX4. Data are presented as mean ± SD, n = 3.

Figure S15.

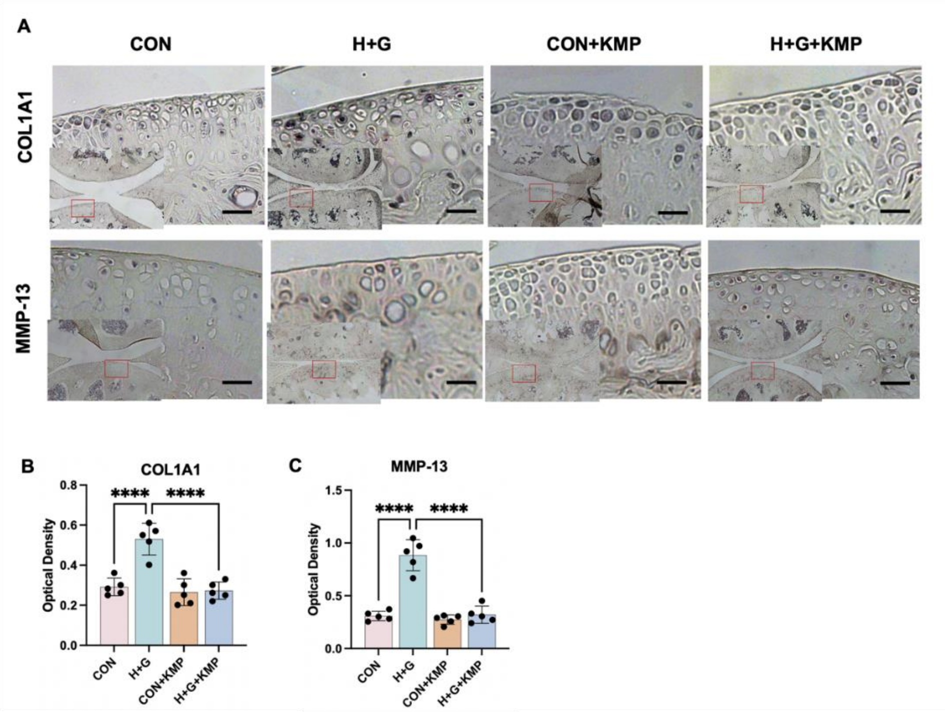

Figure S15. (A) Representative immunohistochemical staining of collagen type I alpha 1 (COL1A1) and matrix metalloproteinase-13 (MMP-13) in knee joint sections from control (CON), simulated microgravity (H+G), CON treated with KMP (CON+KMP), and H+G treated with KMP (H+G+KMP) mice. Red boxes in lower-magnification images indicate the locations of higher-magnification images. (B–C) Semi-quantitative analysis of (B) COL1A1 and (C) MMP-13 staining across the four groups, quantified as optical density. Scale bars: 25 μm. Data are presented as mean ± SD (n = 5–6 mice per group). ns, not significant; ****$p < 0.0001$. *One-way ANOVA followed by Holm-Šídák's multiple comparisons test was performed.*

Table S1. Antibodies used for Immunohistochemistry (IHC) or Western blot (WB).

**Antibody**	**Origin**	**Cat. No**	**Species**	**Assay**	**Dilution**
GAPDH (D16H11) XP® Rabbit mAb #5174	Cell Signaling Technology	5174S	Rabbit	WB	1:1000
Anti-Collagen II antibody	Abcam	ab34712	Rabbit	WB	1:1000
Anti-Collagen II antibody	Abcam	ab34712	Rabbit	IHC	1:200
Anti-MMP13 antibody	Abcam	ab39012	Rabbit	WB	1:1000
Anti- Collagen I antibody	Abcam	ab138492	Rabbit	IHC	1:200
Anti-Collagen X antibody	Abcam	ab182563	Rabbit	IHC	1:200
Anti-NF-kB p65 antibody	Abcam	ab16502	Rabbit	WB	1:1000
Phospho-NF-κB p65 antibody	Cell Signaling Technology	93H1	Rabbit	WB	1:1000
Anti-CDKN2A/p16INK4a antibody	Abcam	ab211542	Rabbit	WB	1:1000
Anti-CDKN2A/p16INK4a antibody	Abcam	ab211542	Rabbit	IHC	1:200
Anti-p21 antibody	Abcam	ab109520	Rabbit	WB	1:1000
Anti-p21 antibody	Abcam	ab109520	Rabbit	IHC	1:300
Anti-TOMM20 antibody	Abcam	ab186735	Rabbit	IHC	1:200
NOX4 Polyclonal antibody	Proteintech	14347-1-AP	Rabbit	WB	1:1000
NOX4 Polyclonal antibody	Proteintech	14347-1-AP	Rabbit	IHC	1:300
Goat Anti-Rabbit IgG H&L (HRP)	Abcam	ab6721	Goat	WB	1:5000

Table S2. Primers for qRT-PCR

Gene	Forward primer (5'-3')	Reverse primer (5'-3')
SOX9	GGCGGAGGAAGTCGGTGAAGAA	GGCGGACAGGCCCTTCT
COL2A1	GGATGGCTGCACGAAACATACCGG	CAAGAAGCAGACCGGCCCTATG
ADAMTS4	GAGGAGGAGATCGTGTTTCCA	CCAGCTCTAGTAGCAGCGTC
ADAMTS5	GAACATCGACCAACTCTACTCCG	CAATGCCCACCGAACCATCT
NF-KB	AACAGAGAGGATTTCGTTTCCG	TTTGACCTGAGGGTAAGACTTCT
MMP13	ATGCAGTCTTTCTTCGGCTTAG	ATGCCATCGTGAAGTCTGGT
ACAN	AGTCACACCTGAGCAGCATC	AGTTCTCAAATTGCATGGGGTGTC
IL-6	ACTCACCTCTTCAGAACGAATTG	CCATCTTTGGAAGGTTCAGGTTG
IL-8	TTTTGCCAAGGAGTGCTAAAGA	AACCCTCTGCACCCAGTTTTC
RPL13A	GCCATCGTGGCTAAACAGGTA	GTTGGTGTTCATCCGCTTGC
MT-CO1	CAGCAGTCCTACTTCTCCTATCTCT	GGGTCGAAGAAGGTGGTGTT
MT-CO2	GCCCTTTTCCTAACACTCACAACAA	GTAAAGGATGCGTAGGGATGGG
MT-ND1	CCCTAAAACCCGCCACATCT	GGCTAGAATAAATAGGAGGCCTAGGT
MT-RNR1	ATGCAGCTCAAAACGCTTAGC	GCTGGCACGAAATTGACCAA
MT-RNR2	CCCTGTACGAAAGGACAAGAGAAAT	TCTTGGGTGGGTGTGGGTATAAT
PDK1	CTGGCTGTGGCTTCTCTAGC	CCGAAGTCCAGGAACTGCTT
LDHA	TGTCTCTGGCAAAGTGGATATCTT	TGTTCAGTGAAGGAGCCAGG
SLC2A1	TGAGCATCGTGGCCATCTTT	AGGCATGGAACCATTCAGGG
EGLN1	GAAACCATTGGGCTGCTCAT	ACACCTTTTTCACCTTGTAGCA
ADCY3	AGTTCCCGCACAGTTCTAGC	GACCACCACGTAGCAGTCAA
RELA	CCCTTCCAAGAAGAGCAGCG	TCACTCGGCAGATCTTGAGC
RHOA	GAGCCGGTGAAACCTGAAGA	CCCCAGAGCTATGCCAACAA

Table S3. List of gene abbreviations and full gene names

abbreviations	Full name
COL2A1	collagen type II alpha 1 chain
GAPDH	glyceraldehyde-3-phosphate dehydrogenase
ACAN	aggrecan
SOX9	SRY-box transcription factor 9
IL6	interleukin 6
IL8	interleukin 8
ADAMTS4	ADAM metallopeptidase with thrombospondin type 1 motif 4
ADAMTS5	ADAM metallopeptidase with thrombospondin type 1 motif 5
NF-KB	nuclear factor kappa B
MMP13	matrix metallopeptidase 13
p16	cyclin dependent kinase inhibitor 2A
p21	cyclin dependent kinase inhibitor 1A
MMP3	matrix metallopeptidase 3
MMP14	matrix metallopeptidase 14
MMP1	matrix metallopeptidase 1
MMP7	matrix metallopeptidase 7
MMP12	matrix metallopeptidase 12
TNF	tumor necrosis factor
IL-1b	interleukin 1 beta
MT-CO1	mitochondrially encoded cytochrome c oxidase I
MT-CO2	mitochondrially encoded cytochrome c oxidase II
MT-ND1	mitochondrially encoded NADH dehydrogenase 1
MT-RNR1	mitochondrially encoded 12S RNA
MT-RNR2	16S rRNA, mitochondrial
PDK1	pyruvate dehydrogenase kinase 1
LDHA	lactate dehydrogenase A

SLC2A1	solute carrier family 2 member 1
EGLN1	egl-9 family hypoxia inducible factor 1
ADCY3	adenylate cyclase 3
RELA	RELA proto-oncogene, NF-kB subunit
RHOA	ras homolog family member A
NOX4	NADPH oxidase 4
TOMM20	translocase of outer mitochondrial membrane 20
